# Multi-tissue epigenetic analysis identifies distinct associations underlying insulin resistance and Alzheimer’s disease at *CPT1A* locus

**DOI:** 10.1186/s13148-023-01589-4

**Published:** 2023-10-27

**Authors:** Chloé Sarnowski, Tianxiao Huan, Yiyi Ma, Roby Joehanes, Alexa Beiser, Charles S. DeCarli, Nancy L. Heard-Costa, Daniel Levy, Honghuang Lin, Ching-Ti Liu, Chunyu Liu, James B. Meigs, Claudia L. Satizabal, Jose C. Florez, Marie-France Hivert, Josée Dupuis, Philip L. De Jager, David A. Bennett, Sudha Seshadri, Alanna C. Morrison

**Affiliations:** 1 Human Genetics Center, Department of Epidemiology, Human Genetics, and Environmental Sciences, School of Public Health, The University of Texas Health Science Center at Houston, Houston, TX USA; 2 Population Sciences Branch, National Heart, Lung and Blood Institutes of Health, Bethesda, MD USA; 3 Center for Translational and Computational Neuroimmunology, Department of Neurology, Columbia University Irving Medical Center, New York, NY USA; 4 The Framingham Heart Study, Framingham, MA USA; 5 Department of Biostatistics, School of Public Health, Boston University, Boston, MA USA; 6 Department of Neurology, Boston University School of Medicine, Boston, MA USA; 7 Department of Neurology, University of California, Davis, CA USA; 8 Department of Medicine, University of Massachusetts Chan Medical School, Worcester, MA USA; 9 Division of General Internal Medicine, Massachusetts General Hospital, Boston, MA USA; 10 Department of Medicine, Harvard Medical School, Boston, MA USA; 11 Programs in Metabolism and Medical and Population Genetics, Broad Institute of MIT and Harvard, Cambridge, MA USA; 12 Glenn Biggs Institute for Alzheimer’s and Neurodegenerative Diseases, The University of Texas Health Science Center at San Antonio, San Antonio, TX USA; 13 Department of Population Health Sciences, The University of Texas Health Science Center at San Antonio, San Antonio, TX USA; 14 Center for Genomic Medicine and Diabetes Unit, Massachusetts General Hospital, Boston, MA USA; 15 Department of Population Medicine, Harvard Medical School and Harvard Pilgrim Health Care Institute, Harvard University, Boston, MA USA; 16 Diabetes Unit, Massachusetts General Hospital, Boston, MA USA; 17 Department of Medicine, Université de Sherbrooke, Sherbrooke, QC Canada; 18 Department of Epidemiology, Biostatistics and Occupational Health, School of Population and Global Health, McGill University, Montreal, Canada; 19 Taub Institute for Research on Alzheimer’s Disease and the Aging Brain, Columbia University Irving Medical Center, New York, NY USA; 20 Rush Alzheimer’s Disease Center, Rush University Medical Center, Chicago, IL USA

**Keywords:** Epigenetics, Insulin resistance, Alzheimer’s disease, FHS, ROSMAP, DNA methylation

## Abstract

**Background:**

Insulin resistance (IR) is a major risk factor for Alzheimer’s disease (AD) dementia. The mechanisms by which IR predisposes to AD are not well-understood. Epigenetic studies may help identify molecular signatures of IR associated with AD, thus improving our understanding of the biological and regulatory mechanisms linking IR and AD.

**Methods:**

We conducted an epigenome-wide association study of IR, quantified using the homeostatic model assessment of IR (HOMA-IR) and adjusted for body mass index, in 3,167 participants from the Framingham Heart Study (FHS) without type 2 diabetes at the time of blood draw used for methylation measurement. We identified DNA methylation markers associated with IR at the genome-wide level accounting for multiple testing (*P* < 1.1 × 10^−7^) and evaluated their association with neurological traits in participants from the FHS (*N* = 3040) and the Religious Orders Study/Memory and Aging Project (ROSMAP, *N* = 707). DNA methylation profiles were measured in blood (FHS) or dorsolateral prefrontal cortex (ROSMAP) using the Illumina HumanMethylation450 BeadChip. Linear regressions (ROSMAP) or mixed-effects models accounting for familial relatedness (FHS) adjusted for age, sex, cohort, self-reported race, batch, and cell type proportions were used to assess associations between DNA methylation and neurological traits accounting for multiple testing.

**Results:**

We confirmed the strong association of blood DNA methylation with IR at three loci (cg17901584–*DHCR24*, cg17058475–*CPT1A*, cg00574958–*CPT1A*, and cg06500161–*ABCG1*). In FHS, higher levels of blood DNA methylation at cg00574958 and cg17058475 were both associated with lower IR (*P* = 2.4 × 10^−11^ and *P* = 9.0 × 10^–8^), larger total brain volumes (*P* = 0.03 and *P* = 9.7 × 10^−4^), and smaller log lateral ventricular volumes (*P* = 0.07 and *P* = 0.03). In ROSMAP, higher levels of brain DNA methylation at the same two *CPT1A* markers were associated with greater risk of cognitive impairment (*P* = 0.005 and *P* = 0.02) and higher AD-related indices (CERAD score: *P* = 5 × 10^−4^ and 0.001; Braak stage: *P* = 0.004 and *P* = 0.01).

**Conclusions:**

Our results suggest potentially distinct epigenetic regulatory mechanisms between peripheral blood and dorsolateral prefrontal cortex tissues underlying IR and AD at *CPT1A* locus.

**Supplementary Information:**

The online version contains supplementary material available at 10.1186/s13148-023-01589-4.

## Background

Alzheimer’s disease (AD) is a progressive neurodegenerative disorder and the most common form of age-related dementia. While aging is clearly the strongest AD risk factor, emerging data suggest that type 2 diabetes (T2D), a chronic peripheral metabolic disorder, can contribute substantially to AD pathogenesis or progression, either directly or as a cofactor [1]. Patients with T2D are at higher risk of developing mild cognitive impairment (MCI), all-cause or AD dementia, and have more rapid progression of AD [2–4]. AD itself is associated with increased T2D prevalence and may represent a form of diabetes that selectively affects the brain and has molecular and biochemical features that overlap with T2D [1–5].

Midlife obesity, defined by body mass index (BMI) higher than 30 kg/m^2^, is another major risk factor for AD [6]. One pathologic feature shared by T2D and obesity is insulin resistance (IR), a reduced sensitivity in body tissues to insulin action. Insulin is produced by pancreatic β cells, circulate in blood to act at its target organs (e.g., liver, muscle), and can be transported into the cerebrospinal fluid to act in the central nervous system. Brain IR, the failure of brain cells (neurons and glial cells) to respond optimally to insulin, is an early, common and major feature in patients with AD [7, 8], whether they have diabetes or not [7, 9, 10]. It is also a major risk factor for subsequent development of AD [11, 12], with evidence for central IR in non-diabetic AD brains [10, 13], a dysregulated glucose metabolism and peripheral IR in patients with AD who do not have diabetes.

Large-scale genetic studies have successfully identified genetic variants associated with T2D, AD and related traits (such as glycemic traits or brain volumes) [14–17]. Some associated genetic variants, genes or regions are shared between AD and T2D [18]. A limited number of studies attempted to jointly analyze T2D and AD or related traits. A cross-trait analysis of metabolic traits and AD identified, for example, a genetic association between glycemic traits (including fasting insulin) and AD and a few shared genetic loci, thus providing insights into the underlying shared genetic architecture between IR and AD [19]. However, the genetic variants identified by genetic studies are often common in the population with relatively modest effect sizes and explain a limited proportion of the variance or heritability of the traits/diseases studied. Thus, additional mechanisms related to gene regulation, such as epigenetic marks, need to be considered to dissect and improve our understanding of the biological and regulatory mechanisms involved in IR and AD.

Epigenetic modifications play a role in the pathogenesis and progression of T2D [20–22] and AD [20, 23]. A number of epigenome-wide association studies (EWAS) successfully identified DNA methylation markers associated with both T2D [24, 25] and AD [26–28]. Mechanisms that affect IR in peripheral tissues in T2D may be implicated in impaired brain insulin signaling in AD [11]. A functional study conducted using animal models showed, for example, that T2D can induce epigenetic modifications in the brain, leading to structural or functional changes that increase the risk of developing neurological disorders, such as AD [29]. Epigenetic studies may thus help identify molecular signatures of IR associated with AD. However, few blood- or brain tissue-based EWAS have been conducted for IR (quantified using the Homeostatic Model Assessment of IR, HOMA-IR) or fasting insulin (FI), and HOMA-IR analyses have been limited in terms of sample sizes [30–36].

Analyzing up to 3,167 participants from the Framingham Heart Study (FHS) with blood DNA methylation data, we propose to follow a two-step approach that consists in: (1) conducting an EWAS of IR, quantified using HOMA-IR and adjusted for BMI, and (2) evaluating the association of IR-associated blood DNA methylation markers with neurological traits (all-cause dementia, AD dementia and brain volumes derived from MRI). In addition, we aim to assess whether the identified DNA methylation markers measured in brain tissue are associated with the clinical diagnosis of cognitive status and AD-related indices using 707 participants from the Religious Orders Study (ROS) and the Rush Memory and Aging Project (MAP).

## Results

### Description of participants

Our epigenetic analyses included a total of 3167 participants from FHS and 707 from ROSMAP, respectively, in our epigenetic analyses (Table 1). FHS participants have a median age of 60 years [51–68yrs] and ROSMAP participants a median age of 88 years [84–90yrs]. Participants from FHS and ROSMAP are predominantly women (55% and 63%, respectively) and most self-identified as non-Hispanic White (100% and 98%, respectively). FHS participants with T2D at the time of blood draw, used to assess glycemic traits and omics measurements, are excluded from all analyses, as described in the Methods section.

**Table 1:** Description of participants included in the epigenetic analyses of insulin resistance (IR) and/or neurological traits

A. Framingham Heart Study (*N* = 3,167)	
*Age at IR measurement*	
Median [25–75pc] Mean (SD)	60 [51–68] 60 (13)
Sex, *N* Females (%)	1757 (55)
BMI, median [25–75pc] Mean (SD)	27.2 [24.2–30.6] 27.8 (5.2)
Self-Reported Race, *N* White, *N* Black (%)	3167 (100), 0 (0)
HOMA-IR (ln), median [25–75pc] Mean (SD)	0.8 [0.4–1.3] 0.8 (0.6)
*Cohort/Substudy,* *N* (%)	
GEN3 Offspring/JHU Offspring/UMN	967 (30.5) 367 (11.6) 1833 (57.9)
All-cause dementia, *N* cases (%)	141 (5)
Alzheimer’s disease dementia, *N* cases (%)	112 (4)
HV, mean (SD)	0.005 (0.0005)
TBV, mean (SD)	0.78 (0.02)
LVV, mean (SD)	0.02 (0.009)

*B. Religious Orders Study (ROS) and Memory and Aging Project (MAP)*, *N* = 707	
*Age at death*	
Median [25–75pc] Mean (SD)	88 [84–90] 86 (5)
Sex, *N* Females (%)	447 (63)
Self-reported Race, *N* White, *N* Black (%)	691 (97.7), 13 (1.8)
Cohort/Substudy, *N* (%) ROS MAP	387 (54.7) 319 (45.1)
*Clinical diagnosis of cognitive status at death*, *N* (%)	
NCI: no cognitive impairment (CI) MCI: mild cognitive impairment, no other condition contributing to CI MCI + : mild cognitive impairment and another condition contributing to CI AD: Alzheimer's dementia, no other condition contributing to CI AD + : Alzheimer's dementia and other condition contributing to CI Other dementia: other primary cause of dementia, no clinical evidence of Alzheimer's dementia	218 (30.8) 159 (22.5) 12 (1.7) 255 (36.1) 44 (6.2) 18 (2.5)
*BRAAK stage: semiquantitative measure of severity of neurofibrillary tangle pathology*, *N* (%)	
0 1: stage I 2: stage II 3: stage III 4: stage IV 5: stage V 6: stage VI	9 (1.3) 57 (8.1) 74 (10.5) 209 (29.6) 199 (28.1) 152 (21.5) 6 (0.8)
CERAD score: semiquantitative measure of neuritic plaques, *N* (%)	
1: definite 2: probable 3: possible 4: no AD	212 (30.0) 238 (33.7) 72 (10.2) 184 (26.0)

*AD* Alzheimer’s Disease/Alzheimer’s Dementia

*IR* Insulin resistance, *HOMA-IR* Homeostatic Model Assessment of IR, *BMI* Body Mass Index, *HV* Hippocampal Volume, *TBV* Total Brain Volume, *LVV* Lateral Ventricular Volume

### Epigenome-wide association analysis of IR

**Fig. 1:**
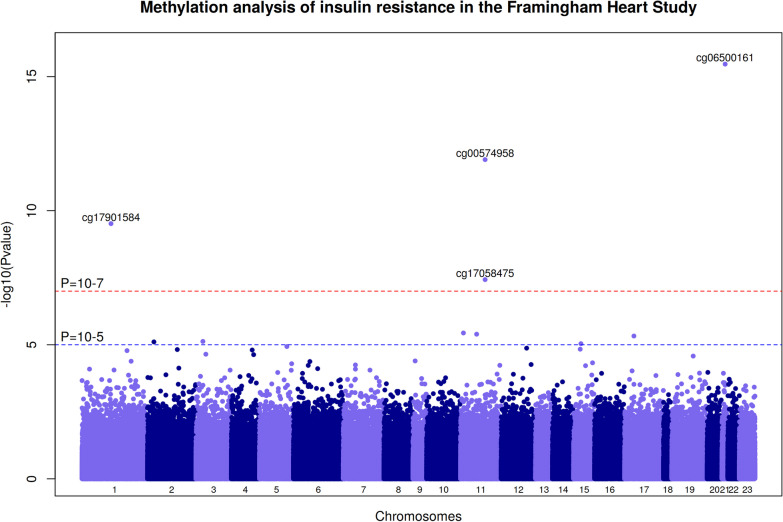
Manhattan plot of the EWAS of HOMA-IR conducted in the Framingham Heart Study. *HOMA-IR* Homeostatic model assessment of insulin resistance. The −log10(P)-value for each blood DNA methylation marker (CpG) on the y axis is plotted against the build 37 genomic position on the x axis (chromosomal coordinate). The dashed horizontal red line indicates the genome-wide significance threshold of *P* = 1.1 × 10^−7^ and the dashed horizontal blue line indicates the threshold of *P* = 10^−5^. Association analysis (*N* = 3167) was adjusted for age, sex, and body mass index

We conduct an EWAS of blood DNA methylation and HOMA-IR in 3,167 FHS participants without T2D. The Manhattan plot is presented in Fig. 1 and the Quantile–Quantile plot in Additional file 1: Figure S1. We detect genome-wide (*P* < 1.1 × 10^−7^) associations between blood DNA methylation and IR at three loci: 1p32 (cg17901584, B = −0.0041, *P* = 2 × 10^−8^, *DHCR24*), 11q13 (cg17058475, B = − 0.0023, *P* = 9 × 10^−8^ and cg00574958, B = -0.0022, *P* = 2.4 × 10^−11^, *CPT1A*), and 21q22 (cg06500161, B = 0.0036, *P* = 4.3 × 10^−18^, *ABCG1*). An additional six DNA methylation markers are associated with IR at a suggestive threshold (*P* < 10^–5^). Association results for the ten DNA methylation markers are presented in Table 2 and Additional file 1: Table S1. Additional adjustments for current smoking or blood cell counts do not change the magnitude or direction of observed association effects between the main DNA methylation markers and HOMA-IR (Additional file 1: Tables S2 and S3). We did not observe sex-differences in association with HOMA-IR (magnitude and direction of effects) for the main DNA methylation markers, except for cg24590708–*MYO5C* and cg11024682–*SREBF1* for which female-specific associations are identified, and cg17901584–*DHCR24* and cg06500161–*ABCG1* for which stronger associations are observed in females (Additional file 1: Table S4).

**Table 2:** Main peripheral blood DNA methylation markers associated (*P* < 10^−5^) with HOMA-IR in the Framingham Heart Study

Locus	CpG marker	Chr	b37 Position	Closest Gene (bp distance)	B	SE	*P*
1p32	cg17901584	1	55,353,706	*DHCR24 (785)*	− 0.0047	0.0007	***3.0* × *10***^***–10***^
2p23	cg15150970	2	25,473,529	*DNMT3A (0)*	0.0023	0.0005	7.8 × 10^–6^
3p22	cg22948094	3	41,172,376	*CTNNB1 (64,025)*	− 0.0038	0.0009	7.5 × 10^–6^
11p15	cg12729894	11	1,774,378	*MOB2/CTSD (0)*	− 0.0028	0.0006	3.6 × 10^–6^
11q12	cg27640302	11	57,296,133	*TIMM10 (0)*	0.0018	0.0004	4.0 × 10^–6^
11q13	cg00574958	11	68,607,622	*CPT1A (0)*	− 0.0024	0.0003	***1.2* × *10***^***–12***^
11q13	cg17058475	11	68,607,737	*CPT1A (0)*	− 0.0025	0.0005	***3.7* × *10***^***–8***^
15q21	cg24590708	15	52,554,357	*MYO5C (0)*	0.0048	0.0011	9.1 × 10^–6^
17p11	cg11024682	17	17,730,094	*SREBF1 (0)*	0.0016	0.0003	4.7 × 10^–6^
21q22	cg06500161	21	43,656,587	*ABCG1 (0)*	0.0035	0.0004	***3.4* × *10***^***–16***^

*IR* Insulin Resistance, *HOMA-IR* Homeostatic Model Assessment of IR. Analyses were conducted in the three batches separately and results were combined using a meta-analysis approach; total *N* = 3167

*P* values in bold and italic passed the multiple-testing significance threshold (*P *< 1.1 × 10^-7^)

Linear mixed-effects models, adjusted for age, sex, and body mass index, were used to evaluate association of blood DNA methylation (outcome) with HOMA-IR

Among the ten DNA methylation markers identified, six are intronic, two are exonic (cg12729894–*CTSD* and cg27640302–*TIMM10*), and two are intergenic (cg17901584–*DHCR24* and cg22948094–*CTNNB1*, the closest gene based on physical distance being used for annotations), Table 2.

### Association analysis of IR-associated blood DNA methylation markers with neurological traits

We evaluate the association of the ten significant or suggestive HOMA-IR blood DNA methylation markers with five neurological traits including all-cause and AD dementia and three brain volumes [hippocampal volume (HV), total brain volume (TBV), and lateral ventricular volume (LVV)] in up to 2279 FHS participants. We further examined associations with three additional neurological traits in 707 ROSMAP participants with brain DNA methylation data, which included one clinical diagnosis at time of death (AD dementia, other dementia, MCI or no cognitive impairment), and two AD-related indices (i.e., Braak stage and CERAD score). Additional information is provided in the Methods section.

In FHS, higher blood DNA methylation at cg17058475 and cg00574958 (both located in *CPT1A*) is significantly or suggestively associated with larger TBV (*P* = 9.7 × 10^−4^ and *P* = 0.03, respectively) and with smaller log LVV (*P* = 0.03 and *P* = 0.07, respectively), Table 3*.* In ROSMAP, higher brain DNA methylation at the same two markers, i.e., cg00574958 and cg17058475 (*CPT1A*), is significantly associated with higher CERAD scores (*P* = 5 × 10^−4^ and *P* = 0.001, respectively), and suggestively with higher Braak stage (*P* = 0.004 and *P* = 0.01, respectively) and greater risk of cognitive impairment (*P* = 0.005 and *P* = 0.02, respectively), Table 4. In FHS, lower blood DNA methylation at both cg12729894 (*MOB2*/*CTSD*) and cg15150970 (*DNMT3A*) is suggestively associated with greater risk of AD (*P* = 0.007), Table 3. In ROSMAP, we observe nominal positive association of cg22948094 (*CTNNB1*) with clinical diagnosis of cognitive status (*P* = 0.02), Table 4. In FHS, the association of the top DNA methylation markers with risk of Alzheimer’s dementia appears similar in terms of magnitude and direction of effects in the two sex strata (Additional file 1: Table S5) except for cg15150970–*DNMT3A* for which a higher effect size and thus a stronger association is observed in the male strata. On the contrary, the association of the top DNA methylation markers with brain volumes appears to differ in terms of magnitude and direction of effects in the two sex strata (Additional file 1: Table S6), with most associations observed in the female strata only. In ROSMAP, the association of the top DNA methylation markers with neurological traits appears similar in terms of magnitude and direction of effects in the two sex strata (Additional file 1: Table S7) We ought to caution in the interpretation of these sex-stratified results due to multiple testing and limited sample sizes.

**Table 3:** Association of 10 IR-associated peripheral blood DNA methylation markers with neurological traits in the Framingham Heart Study

A. all-cause and Alzheimer's dementia									
				All-cause dementia			Alzheimer’s dementia		
CpG marker	Chr	b37 Position	Closest Gene (bp distance)	B	SE	*P*	B	SE	*P*
cg17901584	1	55,353,706	*DHCR24 (785)*	− 0.0016	0.002	0.43	− 0.0039	0.002	0.09
cg15150970	2	25,473,529	*DNMT3A (0)*	− 0.0020	0.001	0.12	− 0.0038	0.001	0.007
cg22948094	3	41,172,376	*CTNNB1 (64,025)*	0.0019	0.002	0.43	0.0025	0.003	0.36
cg12729894	11	1,774,378	*MOB2/CTSD (0)*	− 0.0025	0.002	0.15	− 0.0052	0.002	0.007
cg27640302	11	57,296,133	*TIMM10 (0)*	− 0.0005	0.001	0.62	0.0001	0.001	0.92
cg00574958	11	68,607,622	*CPT1A (0)*	0.0011	0.001	0.27	0.0014	0.001	0.23
cg17058475	11	68,607,737	*CPT1A (0)*	0.0021	0.001	0.07	0.0023	0.001	0.07
cg24590708	15	52,554,357	*MYO5C (0)*	− 0.0024	0.003	0.42	− 0.0049	0.003	0.14
cg11024682	17	17,730,094	*SREBF1 (0)*	− 0.0019	0.001	0.07	− 0.0021	0.001	0.07
cg06500161	21	43,656,587	*ABCG1 (0)*	− 0.0021	0.001	0.09	− 0.0027	0.001	0.05

B. Brain volumes												
				Hippocampal volume			Total brain volume			Log lateral ventricular volume		
CpG marker	Chr	b37 Position	Closest Gene (bp distance)	B	SE	*P*	B	SE	*P*	B	SE	*P*
cg17901584	1	55,353,706	*DHCR24 (785)*	− 0.515	0.943	0.58	− 0.021	0.020	0.28	0.0007	0.0010	0.49
cg15150970	2	25,473,529	*DNMT3A (0)*	0.903	0.626	0.15	0.021	0.013	0.10	− 0.0013	0.0007	0.06
cg22948094	3	41,172,376	*CTNNB1 (64,025)*	− 1.511	1.029	0.14	− 0.012	0.022	0.57	− 0.0014	0.0011	0.21
cg12729894	11	1,774,378	*MOB2/CTSD (0)*	0.793	0.736	0.28	0.023	0.016	0.14	− 0.0013	0.0008	0.10
cg27640302	11	57,296,133	*TIMM10 (0)*	0.369	0.486	0.45	0.012	0.010	0.24	− 0.0002	0.0005	0.68
cg00574958	11	68,607,622	*CPT1A (0)*	− 0.142	0.428	0.74	0.020	0.009	0.03	− 0.0008	0.0005	0.07
cg17058475	11	68,607,737	*CPT1A (0)*	0.291	0.553	0.60	0.038	0.011	***9.7 × 10***^***−4***^	− 0.0013	0.0006	0.03
cg24590708	15	52,554,357	*MYO5C (0)*	0.646	1.330	0.63	0.007	0.028	0.81	− 0.0004	0.0015	0.78
cg11024682	17	17,730,094	*SREBF1 (0)*	0.095	0.445	0.83	0.002	0.009	0.79	− 0.0007	0.0005	0.14
cg06500161	21	43,656,587	*ABCG1 (0)*	0.786	0.549	0.15	0.002	0.012	0.83	− 0.0002	0.0006	0.72

Insulin resistance (IR)

*P* values in bold and italic passed the multiple-testing significance threshold (*P* < 0.001)

Linear mixed-effects models were used to evaluate association of dementia risk (all-cause dementia or Alzheimer’s disease dementia, *N* = 2175) or brain MRI volumes (*N* = 2279) with blood DNA methylation (outcome) while adjusting for difference between age at survival or age at MRI, respectively, with age at DNA methylation measurement, sex, and blood cell counts

**Table 4:** Association of 10 IR-associated peripheral blood DNA methylation markers with neurological traits in ROSMAP

CpG marker	Closest Gene (bp distance)	Clinical diagnosis of cognitive status at time of death			Braak stage						CERAD score						
		B	SE	*P*	B		SE		*P*		B			SE		*P*	
cg17901584	*DHCR24 (785)*	− 0.0006	0.0009	0.50	0.0006		0.0012		0.60		0.0014			0.0012		0.25	
cg15150970	*DNMT3A (0)*	0.0008	0.0007	0.25	0.0002		0.0008		0.78		0.0004			0.0009		0.63	
cg22948094	*CTNNB1 (64,025)*	0.0023	0.0010	0.02	0.0014		0.0012		0.27		− 0.0017			0.0013		0.17	
cg12729894	*MOB2/CTSD (0)*	− 0.0007	0.0005	0.14	− 0.0001		0.0006		0.92		0.0008			0.0006		0.23	
cg27640302	*TIMM10 (0)*	0.0007	0.0006	0.29	0.0004		0.0008		0.62		− 0.0004			0.0008		0.63	
cg00574958	*CPT1A (0)*	0.0013	0.0004	0.005	0.0016		0.0006		0.004		− 0.0020			0.0006		***0.0005***	
cg17058475	*CPT1A (0)*	0.0012	0.0005	0.02	0.0016		0.0006		0.01		− 0.0021			0.0007		***0.001***	
cg24590708	*MYO5C (0)*	0.0011	0.0017	0.52	− 0.0003		0.0022		0.88		0.0015			0.0022		0.50	
cg11024682	*SREBF1 (0)*	− 0.0002	0.0006	0.79	− 0.0002		0.0008		0.79		0.0002			0.0008		0.79	
cg06500161	*ABCG1 (0)*	− 0.0007	0.0006	0.27	3.6 × 10^–5^		0.0008		0.96		0.0013			0.0008		0.10	

*ROSMAP* Religious Orders Study (ROS) and Memory and Aging Project (MAP)

Clinical diagnosis of cognitive status at time of death (6 other dementia, 5 Possible Alzheimer’s dementia (AD), 4 Probable AD, 3 mild cognitive impairment [MCI] and another condition, 2 MCI and no other condition, 1 no cognitive impairment), Braak stage: measure of severity of neurofibrillary tangle pathology (coded 0, I to VI), CERAD score: neuropathologic diagnosis based on estimates of neuritic plaque density (1 definite AD, 2 probable, 3 possible, 4 no AD)

*P* values in bold and italic passed the multiple-testing significance threshold (*P* < 0.002)

Linear regression models were used to evaluate association of brain DNA methylation (outcome) with clinical cognitive diagnosis or AD-related indices (*N* = 707), adjusting for age at death, sex, substudy, self-reported race, batch, and cell type proportions

### Association analysis of blood RNA levels with HOMA-IR in FHS

In FHS, we detect positive associations between blood RNA levels of *CPT1A* (B = 0.02, *P* = 1.9 × 10^−4^), *DHCR24* (B = 0.01, *P* = 0.02), *CTNNB1* (B = [0.01–0.02], *P* = [2.7 × 10^−4^− 0.01]) with HOMA-IR, and negative associations for *ABCG1* (B = -0.04, *P* = 3 × 10^−22^), and *SREBF1* (B = − 0.008, *P* = 9.4 × 10^−4^) with HOMA-IR (Additional file 1: Table S8). We observe inverse effects between the association of DNA methylation and RNA levels with HOMA-IR at a same locus.

### Association of DNA methylation and RNA expression in FHS (blood) and ROSMAP (brain)

In FHS, we observe a strong negative association of blood RNA levels with blood DNA methylation for *CPT1A* (cg00574958: B = − 3.98, *P* = 1.7 × 10^−16^ and cg17058475: B = − 2.05, *P* = 1.2 × 10^−11^), *ABCG1* (cg06500161: B = − 2.39, *P* = 1.2 × 10^−52^), and *DHCR24* (cg17901584: B = − 1.16, *P* = 9.9 × 10^−15^), and a strong positive association for *TIMM10* (cg27640302: B = 4.83, *P* = 5.2 × 10^−33^). A modest negative association is observed for *DNMT3A* (cg15150970: B = − 0.36, *P* = 0.01). No evidence of association is observed for *CTSD*, *CTNNB1, SREBF1* and *MYO5C* probes (Additional file 1: Table S8). In ROSMAP, we detect a negative association of brain RNA expression with brain DNA methylation for *CPT1A* (cg17058475, B = − 0.04, *P* = 0.009), *CTNNB1* (cg22948094, B = − 0.09, *P* = 0.007), and *TIMM10* (cg27640302, B = − 0.02, *P* = 9.6 × 10^−18^) probes (Additional file 1: Table S9).

### Pathway enrichment analysis

We detect significant enrichment for several GO terms, related to lipid, cholesterol, sterol synthesis, storage and transport, mitochondria transport or import as well as amyloid precursor protein (APP) catabolic and metabolic processes (GO:0042987 and GO:0042982), Additional file 1: Tables S10 and S11.

### Expression quantitative trait methylation (eQTMs) and methylation quantitative trait loci (mQTLs)

Using several publicly available resources for mQTLs in the blood (BIOS QTL browser [37], mQTLdb [38], GoDMC browser [39] , and FHS mQTLs results[40]) or in the brain (xQTLServe browser [41]), we identify cis-methylation quantitative trait loci (mQTLs) at cg17901584–*DHCR24*, cg15150970–*DNMT3A*, cg12729894–*CTSD*, cg27640302–*TIMM10*, cg17058475 and cg00574958–*CPT1A*, cg24590708–*MYO5C*, cg11024682–*SREBF1*, and cg06500161–*ABCG1* (Additional file 1: Tables S12–15). In an eQTM analysis conducted in FHS using RNAseq data, strong negative associations were reported for *CPT1A* (cg17058475 and cg00574958), *SREBF1* (cg11024682) and *ABCG1* (cg06500161) DNA methylation markers with blood RNA levels of *CPT1A* and *ABCG1,* respectively, and a strong positive association was reported for *TIMM10* DNA methylation marker (cg27640302) with blood RNA levels of *TIMM10* (Additional file 1: Table S16).

### RNA expression in brain cell types

We present RNA expression profiles in brain cell types from two databases in Additional file 1: Figure S2. *CTNNB1* and *TIMM10* are expressed in most of the brain cells (endothelial, fetal astrocytes, microglia, neurons, oligodendrocytes). *DHCR24* is mainly expressed in fetal astrocytes, neurons, and oligodendrocytes. *DNMT3A* is mainly expressed in fetal astrocytes, and microglia/macrophage. *CPT1A* is mainly expressed in fetal and mature astrocytes and endothelial. *MYO5C* is expressed in endothelial and fetal astrocytes. *SREBF1* is expressed in fetal and nature astrocytes and oligodendrocytes. *ABCG1* is expressed in oligodendrocytes, and microglia/macrophage. *CTSD* is mainly expressed in mature astrocytes, endothelial, and oligodendrocytes.

### Correlation of DNA methylation across tissues (blood cells and brain)

Using the Blood–Brain Epigenetic Concordance (BECon) tool [42] (Additional file 1: Figure S3), we observe modest to high positive correlations of DNA methylation in blood and brain for cg12729894–*CTSD* (Brodmann area (BA) 20, *r* = 0.53), cg27640302–*TIMM10* (BA10, *r* = 0.64), and cg22948094–*CTNNB1* (BA7, *r* = 0.56). Modest to high negative correlations are observed for cg12729894–*CTSD* (BA7, r = − 0.69), cg17058475–*CPT1A* (BA10, r = − 0.64), and cg06500161–*ABCG1* (BA10, *r* = − 0.51). Using the Blood Brain DNA Methylation Comparison Tool [43] from University of Exeter (Additional file 1: Figure S4), we find positive correlations of DNA methylation in blood and brain for cg22948094–*CTNNB1* (prefrontal cortex, *r* = 0.34), cg27640302–*TIMM10* (prefrontal cortex, *r* = 0.29), and cg11024682–*SREBF1* (entorhinal cortex, *r* = 0.24). Using IMAGE-CpG browser[44] (Additional file 1: Table S17), we detect significant positive correlations of DNA methylation in live human brain and blood cells for cg27640302–*TIMM10* (450 K, *r* = 0.75), cg15150970–*DNMT3A* (*r* = 0.53), cg12729894–*CTSD* (*r* = 0.55), cg11024682–*SREBF1* (*r* = 0.46), and cg06500161–*ABCG1* (*r* = 0.75) (EPIC).

## Discussion

By following a two-step epigenetic approach in the FHS, we identified 10 peripheral blood DNA methylation markers associated with HOMA-IR at a significant or suggestive level. Pathway analyses highlighted enrichment of EWAS signals towards ontologies related to mitochondria transport or import, lipid, cholesterol, sterol synthesis, storage and transport and APP catabolic and metabolic processes. By leveraging DNA methylation and gene expression data measured in the same tissue (periphery or brain), we also gained insights into the potential regulatory mechanisms at IR-associated loci. At both cg00574958 and cg17058475–*CPT1A*, cg12729894–*CTSD*, cg22948094–*CTNNB1*, and cg15150970–*DNMT3A*, we detected suggestive or significant associations of DNA methylation levels with AD risk, clinical diagnosis of cognitive status, or AD-related indices in FHS or ROSMAP participants. At these loci, we identified epigenetic associations with IR and AD risk that differed in the periphery and the brain. The function of these genes and their relevance to IR and AD biological pathways is described below.

In FHS, higher levels of blood DNA methylation at both cg00574958 and cg17058475 (*CPT1A*) were associated with lower IR and lower *CPT1A* blood RNA expression [45], and higher *CPT1A* blood RNA levels were positively associated with IR. *CPT1A* encodes the carnitine palmitoyltransferase (CPT) 1A. The CPT system, crucial for the mitochondrial beta-oxidation of long-chain fatty acids, is involved in metabolic syndrome, cardiovascular diseases, T2D, and neurological diseases, including AD [46]. DNA methylation levels at *CPT1A* have been reported associated with many different traits related to IR, including BMI, liver fat, lipids, and T2D by previous EWAS [25, 47–51]. Higher blood DNA methylation levels at *CPT1A* locus were negatively associated with *CPT1A* blood RNA levels and BMI [47, 50]. Blood DNA methylation levels at cg00574958–*CPT1A* have also been reported associated with reduced risk of metabolic diseases, including metabolic syndrome, hypertension, and T2D (Table 5) [25, 52, 53]. Higher levels of blood DNA methylation at both cg00574958 and cg17058475 were associated with higher TBV in FHS and a stronger association was observed in women compared to men for cg17058475. While higher blood DNA methylation levels at *CPT1A* markers were associated with lower IR, higher levels of brain DNA methylation at the same markers were associated with higher values of clinical diagnosis of cognitive status and AD-related indices in ROSMAP, suggesting potentially different and tissue-specific epigenetic regulations at this locus. Stronger and more significant associations were observed in women compared to men, which could be due to a limited power issue in the men strata due to a lower sample size in this subgroup. We did not detect an association of *CPT1A* methylation levels with risk of all-cause or Alzheimer’s Dementia in FHS which could be due to a lack of power given the lower number of AD cases in FHS (4%) compared to ROSMAP (42%). Brain *CPT1A* RNA expression was also associated with lower DNA methylation at cg17058475. CPT activity has been implicated in several neurological and social diseases (Parkinson's disease, AD, and schizophrenia) mainly related to the alteration of the insulin equilibrium in the brain [46] . Integration of blood and brain RNA expression data from ADNI and AMP-AD indicated that CPT1A was involved in the regulation of acylcarnitines and amino acids in AD, and gene co-expression network analysis leveraging AMP-AD brain RNA-seq data suggested the CPT1A-centered subnetwork was associated with neuronal system [54]. CPT1A mRNA levels were found to be increased in the frontal cortex, the temporal cortex and in the parahippocampus gyrus of late onset AD patients compared with nondemented control samples [54, 55].

**Table 5:** Insulin and obesity-related associations reported at the 10 IR-associated blood DNA methylation markers identified in the Framingham Heart Study

CpG marker	Closest gene (bp distance)	CpG–trait association previously reported	Reference (PMIDs)
cg17901584	*DHCR24 (785)*	Fasting insulin (FI), metabolic syndrome, waist circumference (WC), high density lipoprotein cholesterol (HDL-C), triglycerides (TG), body mass index (BMI), hepatic fat	31197173 (negative association), 33239708, 29,762,635, 25935004, 28194238, 28095459, 29278407, 28002404, 28213390, 27350042, 34183656, 30936141
cg15150970	*DNMT3A (0)*	FI	31197173 (positive association)
cg22948094	*CTNNB1 (64,025)*	Not previously reported	
cg12729894	*MOB2/CTSD (0)*	FI	Negative association (identified in a large meta-analysis from CHARGE cohorts – personal communication; not published)
cg27640302	*TIMM10 (0)*	Not previously reported	
cg00574958	*CPT1A (0)*	FI, fasting glucose, BMI, TG, VLDL-C WC, T2D, plasma adiponectin, carbohydrate and fat intake, blood pressure, hepatic fat, metabolic syndrome	31197173 (negative association), 29278407, 28,213,390, 26110892, 32901515, 29099282, 29762635, 25935004, 28173150, 28002404, 24920721, 28194238, 25583993, 33622391, 28139377, 32930325, 29198723, 36345830, 27350042, 34183656, 31506343, 30936141, 26808626
cg17058475	*CPT1A (0)*	FI, BMI, TG, VLDL-C, blood pressure, metabolic syndrome	31197173 (negative association), 29,278,407, 28213390, 24920721, 28194238, 36345830, 27350042, 28095459, 34183656, 26808626
cg24590708	*MYO5C (0)*	FI, BMI driving changes in DNA methylation	31,510,868, 31,197,173 (positive association)
cg11024682	*SREBF1 (0)*	FI, BMI, fasting glucose, WC, T2D, TG, LDL-C, HDL-C, hepatic fat	31197173 (positive association), 29,278,407, 27019061, 28173150, 28213390, 28194238, 25583993, 25935004, 34670603, 29762635, 29099282, 27350042, 34183656, 31506343, 30936141
cg06500161	*ABCG1 (0)*	FI, HOMA-IR, TG, BMI, HDL-C, T2D, hepatic fat	31197173 (positive association), 27019061, 24170695 (positive association), 28213390, 29278407, 28213390, 33239103, 33622391, 28194238, 27350042, 34183656, 31506343, 30936141

*IR* Insulin resistance, *EWAS* Catalog, the MRC–IEU catalog of epigenome-wide association studies [108]

In FHS, higher levels of blood DNA methylation at cg12729894 (*CTSD*) were associated with lower IR and with reduced AD risk. Brain DNA methylation at cg12729894 was not associated with neurological traits in ROSMAP, suggesting a potential periphery-specific epigenetic mechanism underlying IR and AD at this locus. Plasma levels of CTSD (Cathepsin D) have been suggested as a biomarker for IR as they correlate with IR, and are associated with insulin sensitivity and hepatic inflammation [56–59], and have also been reported as a potential diagnostic biomarker for AD and Parkinson's disease [60, 61]. *CTSD* is a good candidate gene for AD [62–64], as it encodes a lysosomal protease important for the degradation of various substrates, including disease-associated proteins, such as α-synuclein (a-syn), amyloid precursor protein (APP) and tau, which tend to aggregate if not efficiently degraded in neurodegenerative disorders [65].

In FHS, higher levels of blood DNA methylation at cg22948094 (*CTNNB1*) were associated with lower IR. Higher levels of *CTNNB1* blood RNA expression were associated with higher IR. To our knowledge, this is the first time this DNA methylation marker is described associated with IR. The catenin beta 1 has high biologic relevance for IR. It is part of the Wnt/β-catenin pathway, that regulates de novo lipogenesis and fatty acid monounsaturation and plays a role in body fat distribution, obesity, metabolic dysfunction, and regulation of adipocyte metabolism [66, 67]. Furthermore, β-catenin mediates effects of Wnt signaling on lipid metabolism in part by transcriptional regulation of Mlxipl and Srebf1. The insulin signaling and the Wnt/β-catenin signaling interact in both peripheral tissues and the brain and may contribute to IR [68]. While higher blood DNA methylation levels at cg22948094 were associated with lower IR, higher levels of brain DNA methylation at cg22948094 were positively associated with clinical diagnosis of impaired cognitive status in ROSMAP, suggesting an epigenetic regulation that could differ across tissues. Higher levels of *CTNNB1* brain RNA expression were also associated with lower DNA methylation. Blood DNA methylation at cg22948094 was not associated with neurological traits in FHS when pooling men and women, but a negative association with LVV was observed in men only. In the brain, the Wnt/β-catenin signaling is crucial for neuronal survival and neurogenesis and is also important to regulate synaptic plasticity and blood–brain barrier integrity and function. Activation of Wnt/β-catenin signaling inhibits amyloid-β production and tau protein hyperphosphorylation in the brain, and a dysregulation in this signaling has been shown to play an important role in AD pathogenesis [69, 70].

In FHS, higher levels of blood DNA methylation at cg15150970 (*DNMT3A*) were associated with higher IR, aligned with previous studies [71], and with reduced AD risk, as found in a recent meta-analysis of blood EWAS of AD conducted in two independent samples (Additional file 1: Table 18), [26] suggesting a potentially different epigenetic mechanism underlying IR and AD in the periphery. In FHS, the association of cg15150970 with AD risk was stronger in men compared to women. Brain DNA methylation at cg15150970 was not associated with neurological traits in ROSMAP. DNA methyltransferase 3 alpha is thought to function in de novo methylation and has been shown to be necessary and sufficient to mediate IR in mouse and human adipocytes [71]. Associations of *DNMT3A* genetic mutations with cognitive decline and late-onset AD risk have also been reported [72, 73].

Among the 10 DNA methylation markers detected associated with IR in FHS, several have been previously reported associated with FI or IR, or with related traits, such as BMI, triglycerides, T2D or metabolic syndrome (Table 5). In addition, blood DNA methylation levels at cg17901584 (*DHCR24*), cg00574958 and cg17058475 (*CPT1A*), cg12729894 (*CTSD*), cg11024682 (*SREBF1*), and cg06500161 (*ABCG1*) have been reported as strongly associated with CAIDE1, [74] a dementia composite risk score calculated using a weighted sum of age, sex, BMI, years in education, systolic blood pressure, and total cholesterol [75]. In a recent meta-analysis combining six EWAS of AD using DNA methylation measured in different brain regions and across cortex [28], DNA methylation levels at both cg00574958 and cg17058475 (*CPT1A*) were reported to be positively associated with AD (Braak stage) in most studies, and similar effects were observed across brain regions (Additional file 1: Figure S5). A cross-tissue meta-analysis of EWAS of AD [26] found a positive association of cg17058475 (*CPT1A*, in both prefrontal cortex and blood) and a negative association of cg15150970 (*DNMT3A,* in blood) with AD risk (Additional file 1: Table S18). Note that these publicly available results did not include all of our main methylation markers. While these results are in line with our findings, it is important to note that we also observed some differences in results between our analysis conducted in ROSMAP and the two publicly available meta-analyses of EWAS of AD mentioned above that both included ROSMAP. We hypothesize that it could be due to difference in sample size, QC, and covariate adjustment. Smith et al*.* opted to derive and adjust analyses for surrogate variables (but not batch) that could be related to disease pathophysiology more than technical or demographic confounders [76].

Strengths of our study include a relatively large sample size, leveraging well-characterized phenotypes and two different blood omics data from two FHS generations, complemented with clinical diagnosis of cognitive status, AD-related indices and omics data measured in the brain (prefrontal cortex) from ROSMAP. Our analyses were conducted in participants predominantly of European ancestry, limiting the generalizability of our results to other population groups. Glycemic traits measurement was not available in ROSMAP, limiting our ability to verify if the brain DNA methylation levels at the main markers identified in the FHS EWAS were associated with brain IR in ROSMAP. We annotated the DNA methylation markers to the closest gene (based on their physical distance to the gene) but acknowledge the possibility that these markers regulate expression of additional genes in the region. Finally, while our results shed light on IR-associated genes with functions relevant to the brain, more studies are needed to fully understand whether the biological mechanisms underlying IR in the periphery and the central nervous system are related or distinct [77].

## Conclusion

Our epigenetic analysis leveraging both blood and brain omics identified potentially distinct epigenetic regulatory mechanisms between the periphery and the dorsolateral prefrontal cortex underlying IR and AD at cg15150970 (*DNMT3A*), cg22948094 (*CTNNB1*), cg12729894 (*CTSD*), cg00574958 and cg17058475 (*CPT1A*). At *CPT1A* locus, higher blood DNA methylation levels at both cg00574958 and cg17058475 were associated with lower IR in the periphery, and higher brain DNA methylation levels at the same markers were associated with increased AD risk. Priority should be given in the future to collect, in the same cohorts, both metabolic and neurological phenotypes, and to measure omics in multiple relevant tissues to advance our understanding of the biological mechanisms involved in IR in both the periphery and the brain.

## Methods

## A. The Framingham Heart Study (FHS)

The FHS is a large population‐based longitudinal study composed of three generations of participants [78–80]. We include in our analyses participants from the second and third generations [the FHS Offspring Cohort who attended the eighth examination cycle (2005–2008) and the FHS Generation 3 Cohort (Gen 3) who attended the second examination cycle (2008–2011)]. All participants provided written informed consent at each examination. The FHS protocol for blood collection and DNA methylation was approved by the Institutional Review Board of the Boston University Medical Campus. This study has been approved by the UTHealth Institutional Review Board (HSC-SPH-21–0072). The FHS data are available on dbGaP (accession number: phs000007.v32.p13). All FHS participants are predominantly Whites/of European descent. Smartpca is used to conduct Principal Component Analysis (PCA) based on genetic data (Affy500K). We exclude ethnic outliers based on 6SD from the mean for the first 10 genetic principal components (PCs).

### Insulin resistance quantification

IR is quantified using HOMA-IR [81], which has been extensively used as validated surrogate of IR, and calculated based on fasting glucose (FG) and FI measurements: HOMA-IR = (FI × FG)/22.5, where FI denotes fasting insulin concentration (mU/l) and FG denotes fasting plasma glucose (mmol/l). HOMA-IR values are log-transformed (natural log) and participants with T2D at IR measurement are excluded from analysis.

### Neurological traits definition

We analyze two main clinical traits: AD dementia and all-cause dementia. The definition for AD dementia is based on clinical criteria (NINCDS–ADRDA) [82]. In addition to clinical definition of dementia disorders, we analyze brain imaging quantitative phenotypes derived from MRI, including total brain (TBV), hippocampal (HV), and lateral ventricular (LVV) volumes, which are endophenotypes for clinical dementia [83]. All measures are obtained through analysis of high-resolution, T1 weighted images acquired as either an MPRAGE or IRSPGR sequence. Skull removal and segmentation of lateral ventricles and hippocampi employs a standard atlas-based diffeomorphic approach [84]. For hippocampal segmentation, the EADC–ADNI harmonized hippocampal masks are used to assure standardization across cohorts [85–87]. After skull removal, a template-based iterative method is used to correct for field inhomogeneity bias [88]. Tissue segmentation is based on an Expectation–Maximization (EM) algorithm that iteratively refines its segmentation estimates to produce outputs that are most consistent with the input intensities from the native-space T1 images along with a model of image smoothness [89, 90]. All brain volumes are adjusted for intracranial volume. LVV values are log-transformed (natural log). Participants with T2D at IR measurement are excluded from analysis.

### Blood DNA methylation data measurement

We use DNA methylation levels measured in blood in the FHS, as such measures were not available in the brain. Peripheral blood samples were collected at the eighth examination for the Offspring Cohort and the second examination for the Gen 3 Cohort. Bisulfite conversion was performed using the EZ DNA Methylation Kit (Zymo Research Corporation, Irvine, CA). Samples underwent DNA amplification, fragmentation, array hybridization, and single-base pair extension. DNA methylation levels quantification was conducted using the Illumina Infinium Human Methylation450 BeadChip (450 K). DNA methylation arrays of the FHS Offspring Cohort participants were run in two laboratory batches at the Johns Hopkins Center for Inherited Disease Research (JHU) and University of Minnesota Biomedical Genomics Center (UMN). DNA methylation arrays of the FHS Gen 3 Cohort participants (GEN3) were run by Illumina (San Diego, CA). Details about DNA methylation measurement and QC in FHS can be found in the Supplement.

### RNA expression measurement

Peripheral blood samples were collected at the eighth examination for the Offspring Cohort and the second examination for the Gen 3 Cohort. Samples from whole blood were collected in PAXgene™ tubes. After RNA amplification, quantification of transcript levels was performed using the Affymetrix Human Exon1.0 ST MicroArray. Transcriptomic data were collected using the robust multi-chip average (RMA) method, as described previously [91–93]. Details about RNA expression measurement and QC in FHS can be found in the Supplement.

## B. The Religious Orders Study (ROS) and the Rush Memory and Aging Project (MAP)

The Religious Orders Study (ROS) is a longitudinal, epidemiologic clinical–pathological study of memory, motor, and functional problems in older Catholic nuns, priests, and brothers from across the United States [76, 94]. Participants without known dementia agree to medical and psychological evaluation and cognitive function testing each year and brain donation after death. All participants sign informed and repository consents and an Anatomic Gift Act. The study was approved by an Institutional Review Board of Rush University Medical Center. Since 1994, more than 1,500 older persons have been enrolled. The methylation data were generated more than a decade ago using all brains available at the time.

The Rush Memory and Aging Project (MAP) is a longitudinal, epidemiologic clinical–pathologic study of dementia and other chronic diseases of aging. Older persons are recruited from about 40 continuous care retirement communities and senior subsidized housing facilities around the Chicago metropolitan area. Participants without known dementia agree to annual detailed clinical evaluation and donation of brain, spinal cord and muscle after death. MAP began in 1997 and over 1600 older adults have enrolled. All participants sign informed and repository consents and an Anatomic Gift Act. The study was approved by an Institutional Review Board of Rush University Medical Center. The methylation data were generated more than a decade ago using all brains available at the time.

We refer in this paper to the joint data set as “ROSMAP”. ROSMAP data were accessed through the AMP-AD Knowledge Portal (synapse ID syn3219045). A short description of sample QC for ROSMAP is available in the Supplement.

### Neurological traits definition

We consider three different neurological traits in ROSMAP, one clinical diagnosis of Alzheimer's dementia, other dementia, MCI or no cognitive impairment (NCI) at time of death, and two AD-related indices: Braak stage and CERAD score [95–100]. Braak Stage is a semiquantitative measure of severity of neurofibrillary tangle (NFT) pathology [97, 99] . CERAD score is a semiquantitative measure of neuritic plaques [99, 100]. Additional details on the trait definitions can be found in the Supplement.

### Brain DNA methylation measurement

Gray matter was dissected from white matter, while on ice from a sample of frozen dorsolateral prefrontal cortex and the cortical sample was processed using the Qiagen QIAamp mini protocol for DNA extraction for each of 761 deceased subjects from the ROS and MAP studies based on the Rush Alzheimer's Disease Center, as previously reported.^86^ Samples were evaporated to increase concentration to 50 ng/ul and submitted to the Broad Institute’s Genomics Platform for processing on the Illumina Infinium HumanMethylation450 BeadChip (450 K). DNA methylation β values reported by the Illumina platform were used as the measurement of methylation level for each CpG probe tagged on the chip. A short description of DNA methylation marker QC for ROSMAP is available in the Supplement. QC for DNA methylation has also been described extensively elsewhere [101, 102].

### RNA expression measurement

We used normalized RNA expression data measured using RNA array from brain samples from Rush University (490 samples, no replicates). cRNA was hybridized to Illumina HT-12 Expression Bead Chip (48,803 transcripts) via standard protocols using an Illumina Bead Station 500GX (Webster et al. 2009). Disease status included 377 with late onset AD, 119 classified with MCI, and 359 healthy non-demented controls. Brain regions sampled in these patients were 726 prefrontal and 129 temporal cortex samples. A short description of RNA expression probes QC for ROSMAP is available in the Supplement. QC for RNA expression data has also been described extensively elsewhere [101, 102].

## C. Association analyses

In FHS, we conduct all association analyses using linear mixed-effects models (GENESIS [103]) and account for familial relatedness using a kinship matrix derived using pedigree information. In ROSMAP, we perform association analyses using linear regression models (lm function) in R.

### Epigenome-wide association analysis (EWAS) of blood cells DNA methylation with IR

In FHS, we evaluate the association between HOMA-IR and blood cells DNA methylation levels (outcome) at all CpGs available after QC (*n* = 441,344). We perform association analyses by batch, adjusting for sex, age at IR and methylation measurement, and BMI. We use METAL [104] to meta-analyse results from the three batches based on an inverse-variance weighted fixed-effects model. Additional adjustment for blood cell counts and current smoking is performed at the top IR-associated blood DNA methylation markers. As sex can strongly influence variation of DNA methylation, additional analyses stratified by sex are conducted for the top EWAS DNA methylation markers. In addition, EWAS results (at *P* < 1.1 × 10^−7^ or *P* < 10^−5^) are used to evaluate enrichment for biological pathways using gene ontology (GO) terms and KEGG pathways and the R package missMethyl [105] with annotations for the 450 K Illumina platform. Enrichment *P* values are adjusted for the number of genes in each pathway.

### Association analysis of IR associated DNA methylation markers with neurological traits

Blood DNA methylation markers associated with HOMA-IR at a significant threshold (*P* < 1.1 × 10^−7^ after applying a Bonferroni correction for the number of CpG sites tested) or at a more liberal and suggestive threshold (*P* < 10^−5^), as CpGs were not all independent, are identified and we evaluate their association with neurological traits in both FHS and ROSMAP using linear-mixed effects (GENESIS) or linear models (lm function in R), respectively. In FHS, we evaluated association of all-cause dementia or AD dementia risk with blood DNA methylation (outcome) while adjusting for sex, blood cell counts, and the absolute difference between age at survival and age at DNA methylation measurement. For brain volumes of FHS participants, we evaluate the association of each brain volume with blood DNA methylation markers while adjusting for sex, blood cell counts, and the absolute difference between age at MRI and age at DNA methylation measurement. In ROSMAP, participants with age at death > 90 years are censored and an age of 90 years is assigned to these participants for analysis. We evaluate the association of brain DNA methylation markers (outcome) with clinical diagnosis of cognitive status and AD-related indices using linear regression models adjusted for age at death, sex, sub-study (ROS and MAP), self-reported race, batch, and neuronal proportions. For both FHS and ROSMAP, we also analyze men and women separately. We define significant associations using *P*_FHS_ < 0.05/10/5 = 0.001 and *P*_ROSMAP_ < 0.05/10/3 = 0.002, to account for the number of DNA methylation markers and phenotypes tested.

### Association analyses with RNA expression levels

We assess in FHS the association of blood RNA levels with HOMA-IR, for the nearest genes from CpGs identified in the EWAS of blood DNA methylation markers with HOMA-IR, using the same model and covariates as used in the EWAS. In addition, we evaluate in both studies (FHS and ROSMAP) the association of RNA expression (measured in blood or brain) with DNA methylation, at the top HOMA-IR EWAS loci using similar model as used in the EWAS.

### Publicly available data lookup

We evaluate whether the DNA methylation markers identified in the EWAS of blood DNA methylation with HOMA-IR were previously reported associated with AD by EWAS performed using blood or brain DNA methylation [26, 28, 105]. We explore expression quantitative trait methylation (eQTMs) loci and DNA methylation quantitative trait loci (mQTLs) at DNA methylation markers of interest [37–41, 45] and RNA expression in brain cell types [106, 107] for the nearest genes of the CpG sites identified in the EWAS of blood DNA methylation and HOMA-IR. Finally, we check the correlation of DNA methylation levels between brain and blood tissues at the main EWAS of HOMA-IR DNA methylation markers.[42–44].

## Web resources

The AMP-AD Knowledge Portal: https://adknowledgeportal.synapse.org/. 

BIOS QTLdb: https://molgenis26.gcc.rug.nl/downloads/biosqtlbrowser/. 

mQTLdb: http://www.mqtldb.org/.

GoDMC: http://mqtldb.godmc.org.uk/index. 

xQTLServe: https://mostafavilab.stat.ubc.ca/xQTLServe/.

Cell type RNA expression: http://celltypes.org/brain/.

Brain RNA seq: https://www.brainrnaseq.org/.

EWAS of AD, MetaAna: https://epigenetics.essex.ac.uk/shiny/MetaAna/. 

EWAS Catalog: http://www.ewascatalog.org/.

BECon: https://redgar598.shinyapps.io/BECon/.

Blood Brain DNA Methylation Correlation: https://epigenetics.essex.ac.uk/bloodbrain/.

The RADC Research Resource Sharing Hub: https://www.radc.rush.edu/documentation.htm

### Supplementary Information

**Additional file 1.** Supplementary Materials including additional Text, Figures and Tables.

## Data Availability

The FHS data are available on dbGaP (accession number: phs000007.v32.p13). The ROSMAP data are available on the AMP-AD Knowledge Portal (synapse ID syn3219045). ROSMAP resources and additional phenotypic data can be requested at https://www.radc.rush.edu.
