# Supplementary material for: Multi-tissue epigenetic analysis identifies distinct associations underlying insulin resistance and Alzheimer’s disease at CPT1A locus
Source: Clin Epigenetics. 2023 Oct 27;15:173. doi: 10.1186/s13148-023-01589-4 (PMC10612362; doi:10.1186/s13148-023-01589-4)
Supplement: Supplementary file 1 — Additional file 1. Supplementary Materials including additional Text, Figures and Tables. [file 13148_2023_1589_MOESM1_ESM.docx]

**Multi-tissue epigenetic analysis identifies distinct associations underlying insulin resistance and Alzheimer’s Disease at *CPT1A* locus**

Table of Contents

[**Supplementary Methods** 1](#_Toc140238382)

[**Supplementary Figures** 7](#_Toc140238383)

[**Supplementary Tables** 17](#_Toc140238384)

# **Supplementary Methods**

The Framingham Heart Study (FHS) (1-3)

Generation of DNA methylation β values

DNA methylation β values were generated using the minfi package,(4)and represent the cellular average methylation level ranging from 0 (fully unmethylated) to 1 (fully methylated). For each batch, the raw β values were background corrected and normalized using the DASEN methodology implemented in the WateRmelon R-package. (5) Any β scores with an associated detection P-value (the probability that the total intensity for a given probe falls within the background signal intensity) greater than 0.01 were removed. The output β values for each DNA methylation marker (CpG) were used in downstream analysis.

Quality Control (QC) at the sample level

Samples with the following criteria were removed: missing rate > 1% (missing methylation values, detection P>0.01 at >1% CpGs), poor matching to the SNP control probe locations, and outliers from multi-dimensional scaling techniques.

QC at the blood DNA methylation marker (CpG) level

DNA methylation markers with the following criteria were removed: missing rate > 20% (missing methylation values, detection P > 0.01 at >20% samples), previously identified to map to multiple locations on sex chromosomes, with an underlying SNP (minor allele frequency > 5% in European Ancestry 1000 Genomes Project) at the CpG site or within 10 bp of the single-base extension.

We imputed missing DNA methylation beta values using a k-nearest neighbor approach (k=10), and derived surrogate variables (SVs), by batch, using design matrices that included age, sex, blood cell counts, +/- IR (Gen 3 N=99 SVs, Offspring JHU N=88 SVs, Offspring UMN N=322 SVs). We used measured blood cell counts when available (Gen 3) and imputed them when not (Offspring), based on raw DNA methylation data using the minfi package.(4) We calculated residuals by regressing the DNA methylation beta values on age, sex, and batch-specific SVs associated with IR at *P*<0.1 (Gen 3 N=27 SVs, Offspring JHU N=12 SVs, Offspring UMN N=46 SVs). Finally, we removed, by batch, DNA methylation markers with low variability (SD<0.0006).

QC at the RNA Expression probe level

We downloaded from dbGaP the QC’ed RNA expression data and the imputed blood cell counts derived using partial least squares predictions based on RNA expression data.(6) Extensive details on RNA expression data QC and blood cell counts imputation is available elsewhere.(6)

Briefly, residuals were obtained by adjusting transcript levels for 13 technical covariates that were found to influence transcript levels, including: mean expression and standard deviation across all probesets, negative control probe mean and standard deviation, positive control probe mean and standard deviation, mean relative log expression across all probesets, median absolute deviation mean across all probesets, mean expression across mismatched probe, the first principal component of the gene expression data (corresponding to laboratory batch), sex, age, and plate batch (as a random effect). Samples of poor RNA quality and outlying mean relative log expression across all probesets or outlying principal component values were removed.

We used predicted blood cell counts available on dbGaP, including white blood cells, platelets, lymphocytes, monocytes, eosinophils, and basophils. These predictions were obtained using partial least squares regression trained on transcript levels and complete blood counts measured in 2,431 Third Generation samples.

The Religious Orders Study (ROS) and the Rush Memory and Aging Project (MAP) Study (7,8)

Neurological traits definition

Clinical diagnosis of dementia and clinical Alzheimer’s dementia were based on criteria of the joint working group of the National Institute of Neurological and Communicative Disorders and Stroke and the Alzheimer’s Disease and Related Disorders Association (NINCDS/ADRDA).(9-11) Diagnosis of MCI was rendered for persons who were judged to have cognitive impairment by the neuropsychologist but judged to not meet criteria for dementia by the clinician. Participants without dementia or MCI were categorized as having no cognitive impairment.

Braak Stage is a semiquantitative measure of severity of neurofibrillary tangle (NFT) pathology.(12,13) Bielschowsky silver stain was used to visualize NFTs in the frontal, temporal, parietal, entorhinal cortex, and the hippocampus. Braak stages were based upon the distribution and severity of NFT pathology: Braak stages I and II indicate NFTs confined mainly to the entorhinal region of the brain; Braak stages III and IV indicate involvement of limbic regions such as the hippocampus; Braak stages V and VI indicate moderate to severe neocortical involvement.

CERAD score is a semiquantitative measure of neuritic plaques.(13,14) A neuropathologic diagnosis was made of no AD, possible AD, probable AD, or definite AD based on semiquantitative estimates of neuritic plaque density as recommended by the Consortium to Establish a Registry for Alzheimer’s Disease (CERAD), modified to be implemented without adjustment for age and clinical diagnosis. A CERAD neuropathologic diagnosis of AD required moderate (probable AD) or frequent neuritic plaques (definite AD) in one or more neocortical regions.

Sample, brain DNA methylation marker, and RNA expression probes QC

Good quality probes were selected according to the detection P value<0.01 across all samples. Probes predicted to cross-hybridize with the sex chromosomes and those having overlaps with known SNP with MAF ≥0.01 (±10 bp) based on the 1000 Genomes database were excluded. At the participant level, PCA based on 50 000 randomly selected probes was used to select participants within ±3 SD from the mean for the 3 first PCs. Participants with poor bisulfite conversion efficiency were removed. Missing β values were imputed using a k-nearest neighbor algorithm (k=100). QC for brain DNA methylation and RNA expression data has been described previously.(15,16) We estimated neuronal proportions based on DNA methylation data using the R package CETS.(17)

**References**

(1) Dawber TR, Kannel WB. The Framingham study. An epidemiological approach to coronary heart disease. Circulation 1966 Oct;34(4):553-555.

(2) Feinleib M, Kannel WB, Garrison RJ, McNamara PM, Castelli WP. The Framingham Offspring Study. Design and preliminary data. Prev Med 1975 Dec;4(4):518-525.

(3) Splansky GL, Corey D, Yang Q, Atwood LD, Cupples LA, Benjamin EJ, et al. The Third Generation Cohort of the National Heart, Lung, and Blood Institute's Framingham Heart Study: design, recruitment, and initial examination. Am J Epidemiol 2007 Jun 1;165(11):1328-1335.

(4) Aryee MJ, Jaffe AE, Corrada-Bravo H, Ladd-Acosta C, Feinberg AP, Hansen KD, et al. Minfi: a flexible and comprehensive Bioconductor package for the analysis of Infinium DNA methylation microarrays. Bioinformatics 2014 May 15;30(10):1363-1369.

(5) Pidsley R, Y Wong CC, Volta M, Lunnon K, Mill J, Schalkwyk LC. A data-driven approach to preprocessing Illumina 450K methylation array data. BMC Genomics 2013 May 1;14:293-293.

(6) Joehanes R, Zhang X, Huan T, Yao C, Ying S, Nguyen QT, et al. Integrated genome-wide analysis of expression quantitative trait loci aids interpretation of genomic association studies. Genome Biol 2017 Jan 25;18(1):16-6.

(7) Bennett DA, Buchman AS, Boyle PA, Barnes LL, Wilson RS, Schneider JA. Religious Orders Study and Rush Memory and Aging Project. J Alzheimers Dis 2018;64(s1):S161-S189.

(8) De Jager PL, Ma Y, McCabe C, Xu J, Vardarajan BN, Felsky D, et al. A multi-omic atlas of the human frontal cortex for aging and Alzheimer's disease research. Sci Data 2018 Aug 7;5:180142.

(9) Bennett DA, Wilson RS, Schneider JA, Evans DA, Beckett LA, Aggarwal NT, et al. Natural history of mild cognitive impairment in older persons. Neurology 2002 Jul 23;59(2):198-205.

(10) Bennett DA, Schneider JA, Aggarwal NT, Arvanitakis Z, Shah RC, Kelly JF, et al. Decision rules guiding the clinical diagnosis of Alzheimer's disease in two community-based cohort studies compared to standard practice in a clinic-based cohort study. Neuroepidemiology 2006;27(3):169-176.

(11) Schneider JA, Arvanitakis Z, Bang W, Bennett DA. Mixed brain pathologies account for most dementia cases in community-dwelling older persons. Neurology 2007 Dec 11;69(24):2197-2204.

(12) Braak H, Braak E. Neuropathological stageing of Alzheimer-related changes. Acta Neuropathol 1991;82(4):239-259.

(13) Bennett DA, Schneider JA, Arvanitakis Z, Kelly JF, Aggarwal NT, Shah RC, et al. Neuropathology of older persons without cognitive impairment from two community-based studies. Neurology 2006 Jun 27;66(12):1837-1844.

(14) Mirra SS, Heyman A, McKeel D, Sumi SM, Crain BJ, Brownlee LM, et al. The Consortium to Establish a Registry for Alzheimer's Disease (CERAD). Part II. Standardization of the neuropathologic assessment of Alzheimer's disease. Neurology 1991 Apr;41(4):479-486.

(15) De Jager PL, Srivastava G, Lunnon K, Burgess J, Schalkwyk LC, Yu L, et al. Alzheimer's disease: early alterations in brain DNA methylation at ANK1, BIN1, RHBDF2 and other loci. Nat Neurosci 2014 Sep;17(9):1156-1163.

(16) Zhang B, Gaiteri C, Bodea L, Wang Z, McElwee J, Podtelezhnikov AA, et al. Integrated systems approach identifies genetic nodes and networks in late-onset Alzheimer's disease. Cell 2013 Apr 25;153(3):707-720.

(17) Guintivano J, Aryee MJ, Kaminsky ZA. A cell epigenotype specific model for the correction of brain cellular heterogeneity bias and its application to age, brain region and major depression. Epigenetics 2013 Mar;8(3):290-302.

(18) van Iterson M, van Zwet EW, BIOS Consortium, Heijmans BT. Controlling bias and inflation in epigenome- and transcriptome-wide association studies using the empirical null distribution. Genome Biol 2017 Jan 27;18(1):19-9.

(19) Amena Keshawarz, Helena Bui, Roby Joehanes, Jiantao Ma, Chunyu Liu, Tianxiao Huan, et al. Expression quantitative trait methylation analysis elucidates gene regulatory effects of DNA methylation: The Framingham Heart Study. medRxiv 2022:2022.04.13.22273839.

# **Supplementary Figures**

**Supplementary Figure** **1**: Quantile-Quantile plot of the epigenome-wide association study of insulin resistance (HOMA-IR) conducted in the Framingham Heart Study using blood DNA methylation (450K)


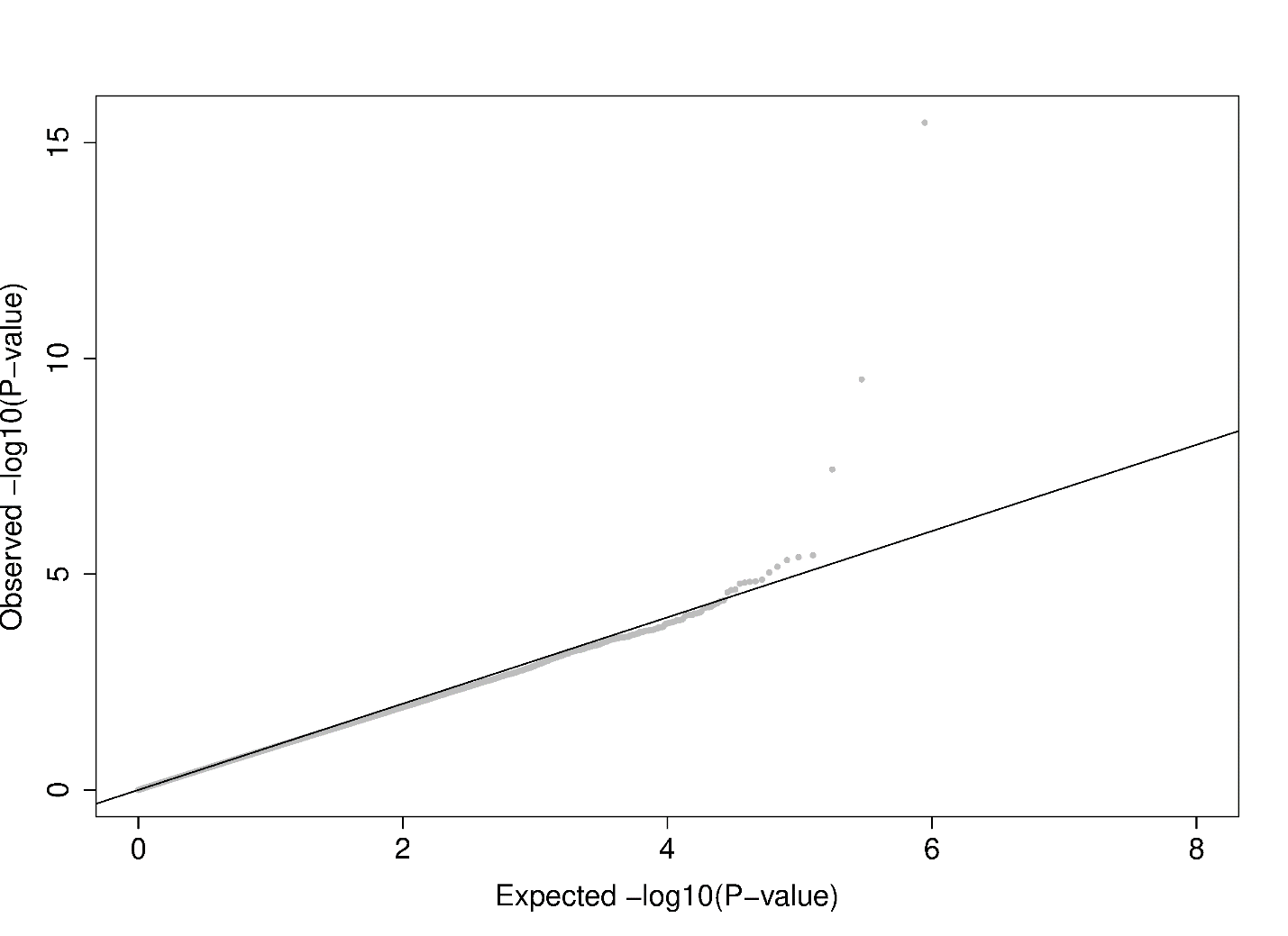


λ_GC_=0.97

**Supplementary Figure 2:** RNA expression in brain cell types of main genes identified in the EWAS of IR conducted in FHS (Figures from <http://celltypes.org/brain/> & <https://www.brainrnaseq.org/>)


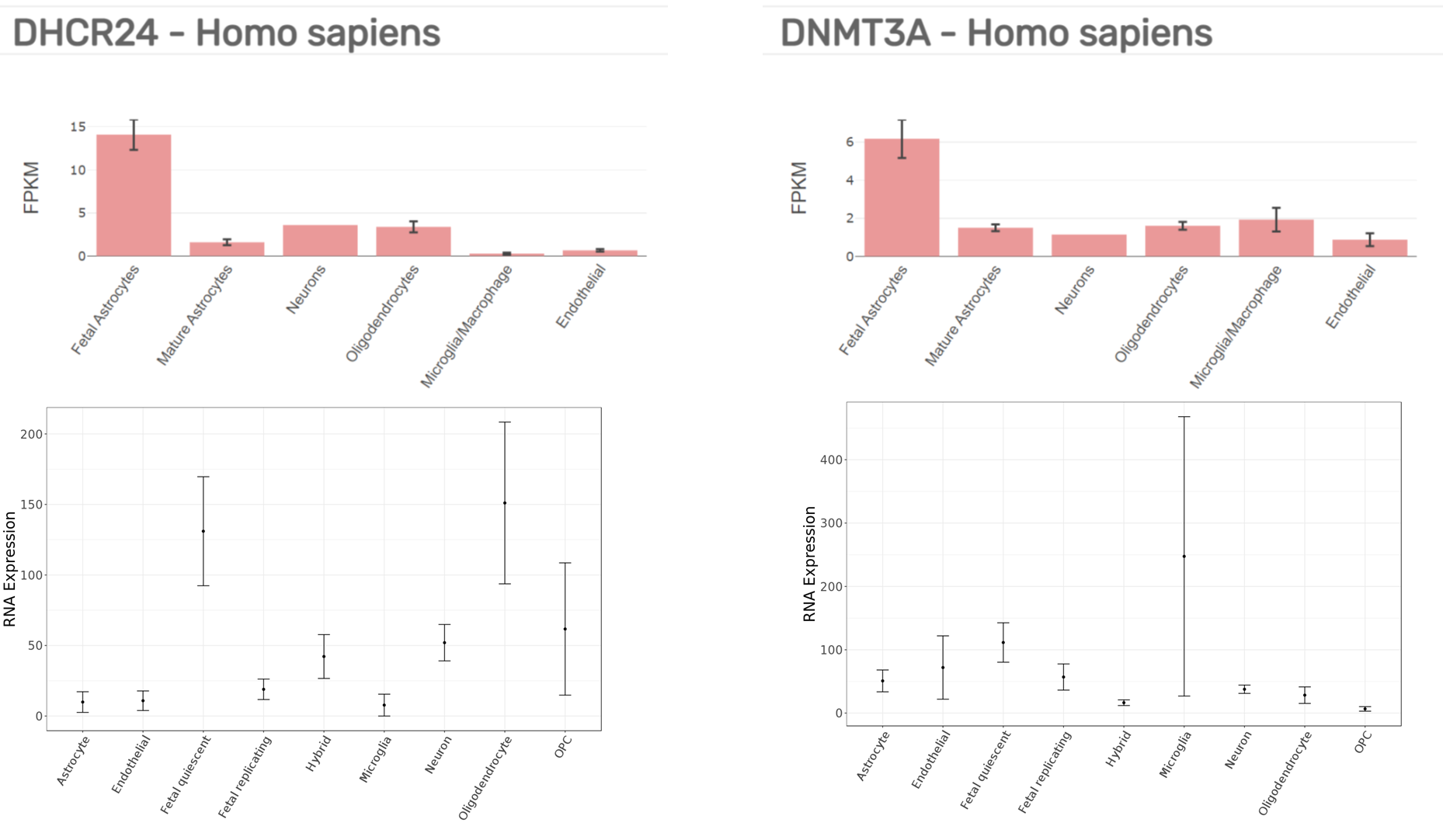


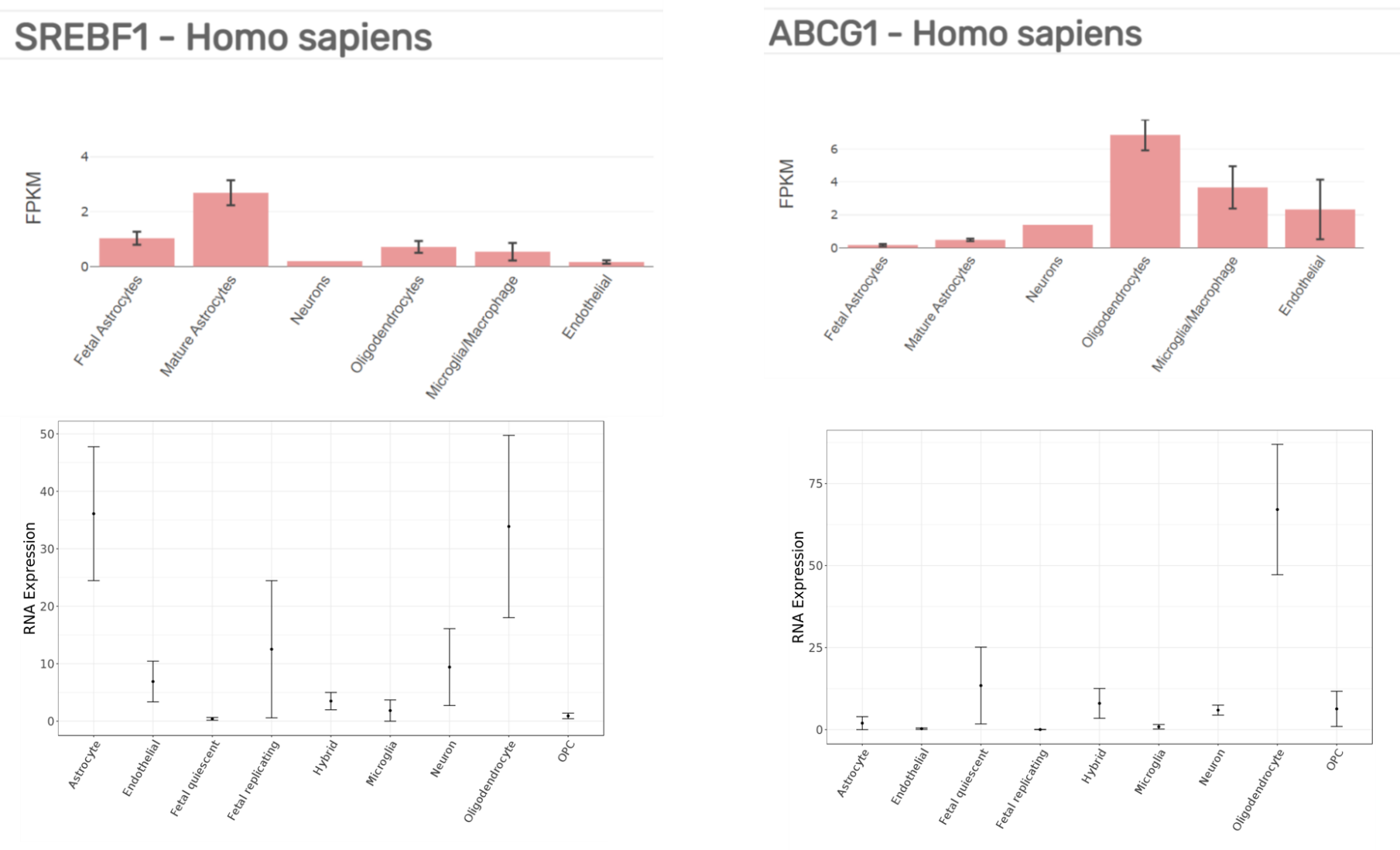

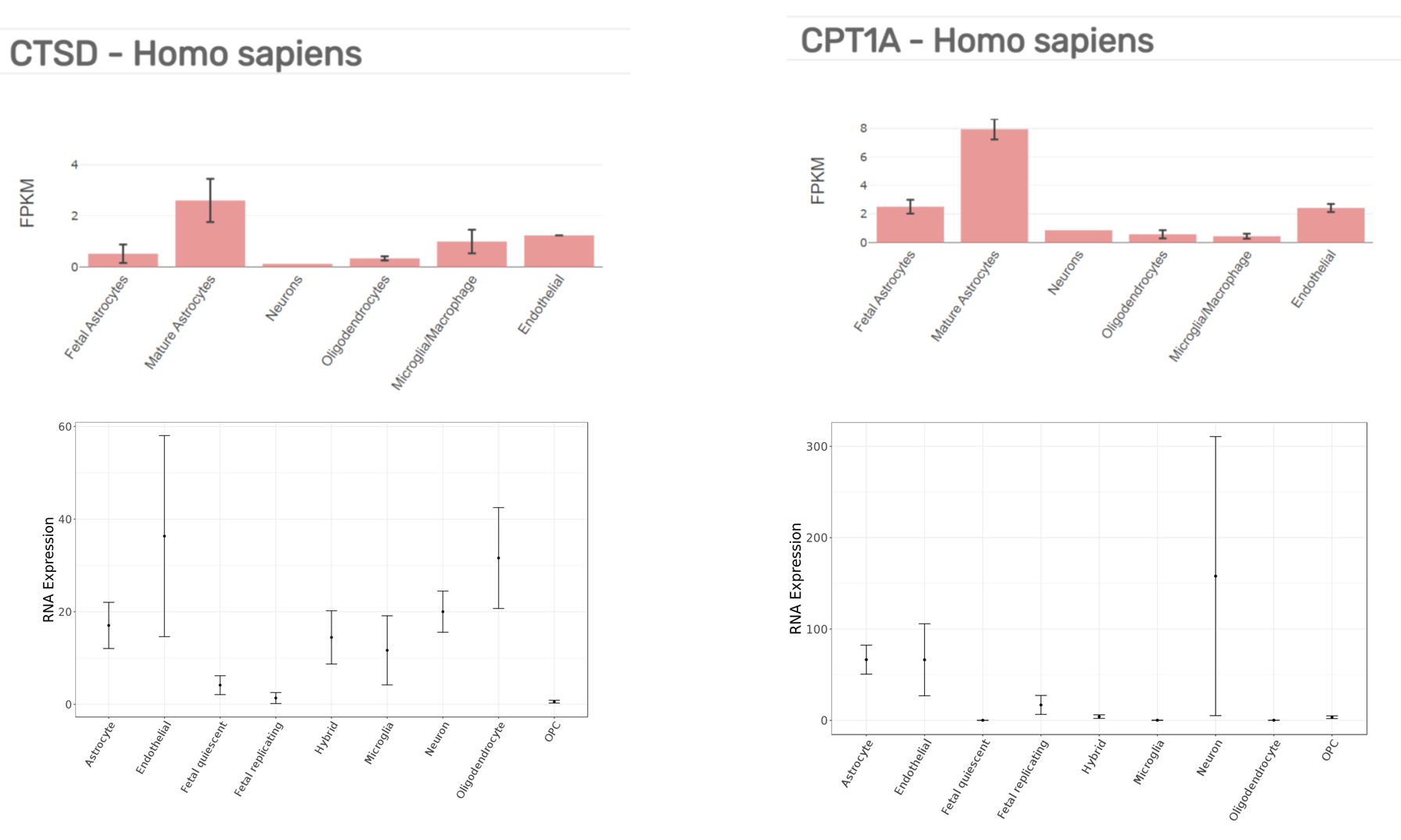

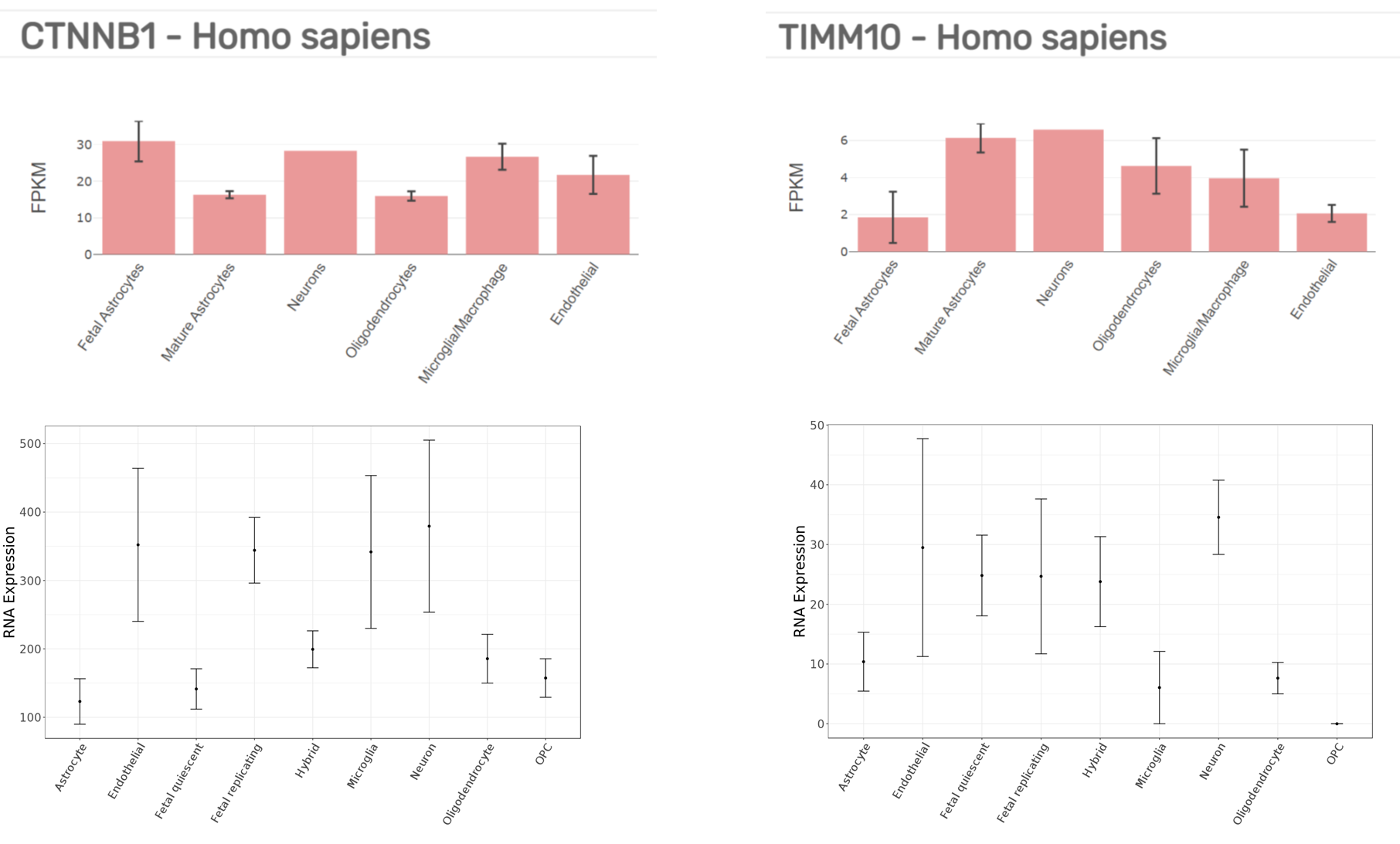

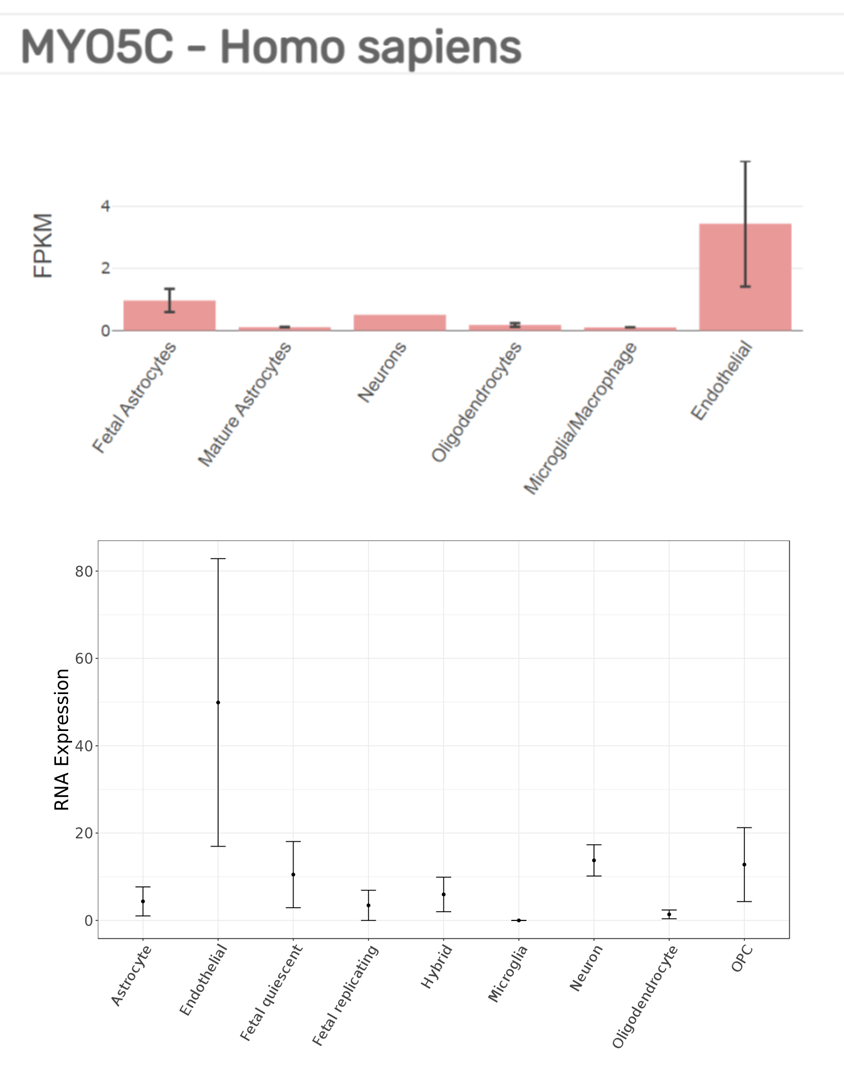


**Supplementary Figure 3:** Correlation of DNA methylation in blood and brain for the main IR-associated DNA methylation markers (Figures from <https://redgar598.shinyapps.io/BECon/>, PMID: 28763057)

**
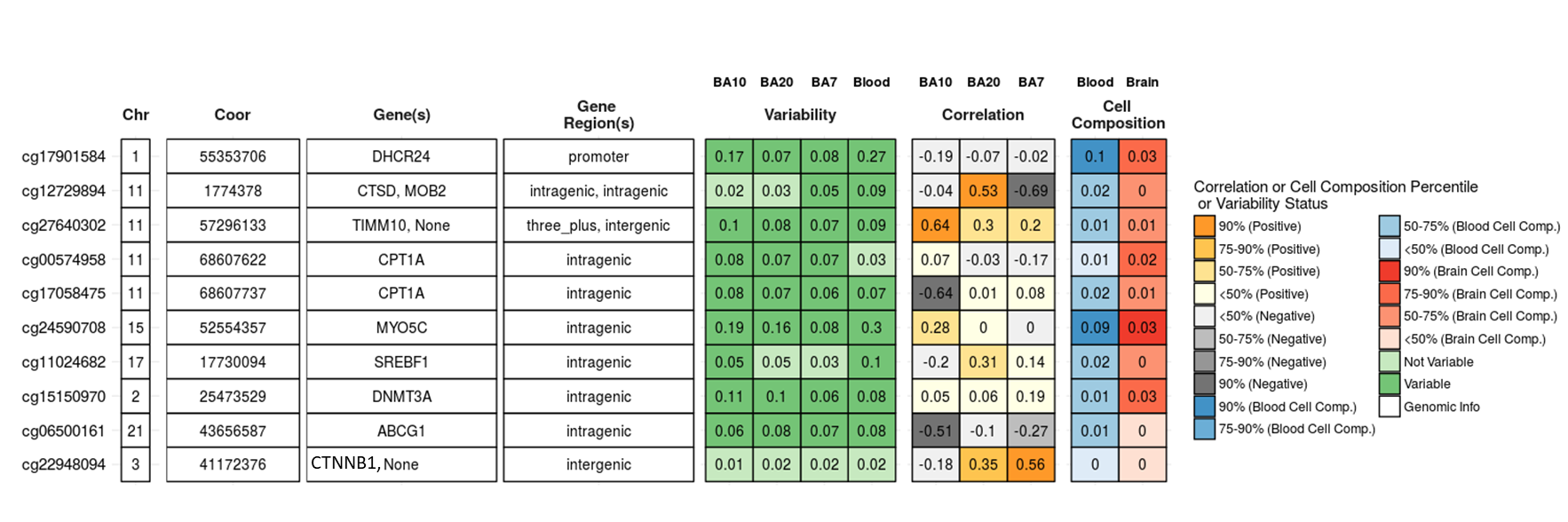
** BECon: Blood–Brain Epigenetic Concordance (<https://redgar598.shinyapps.io/BECon/>)

Paired samples from 16 individuals from three brain regions and whole blood, run on the Illumina 450 K Human Methylation Array

Brain tissue was obtained from the Douglas-Bell Canada Brain Bank

BA refers to Brodmann Areas

**Supplementary Figure 4:** Correlation of DNA methylation in blood and brain for the main IR-associated DNA methylation markers (Figures from: <https://epigenetics.essex.ac.uk/bloodbrain/>, PMID: 26457534)

**
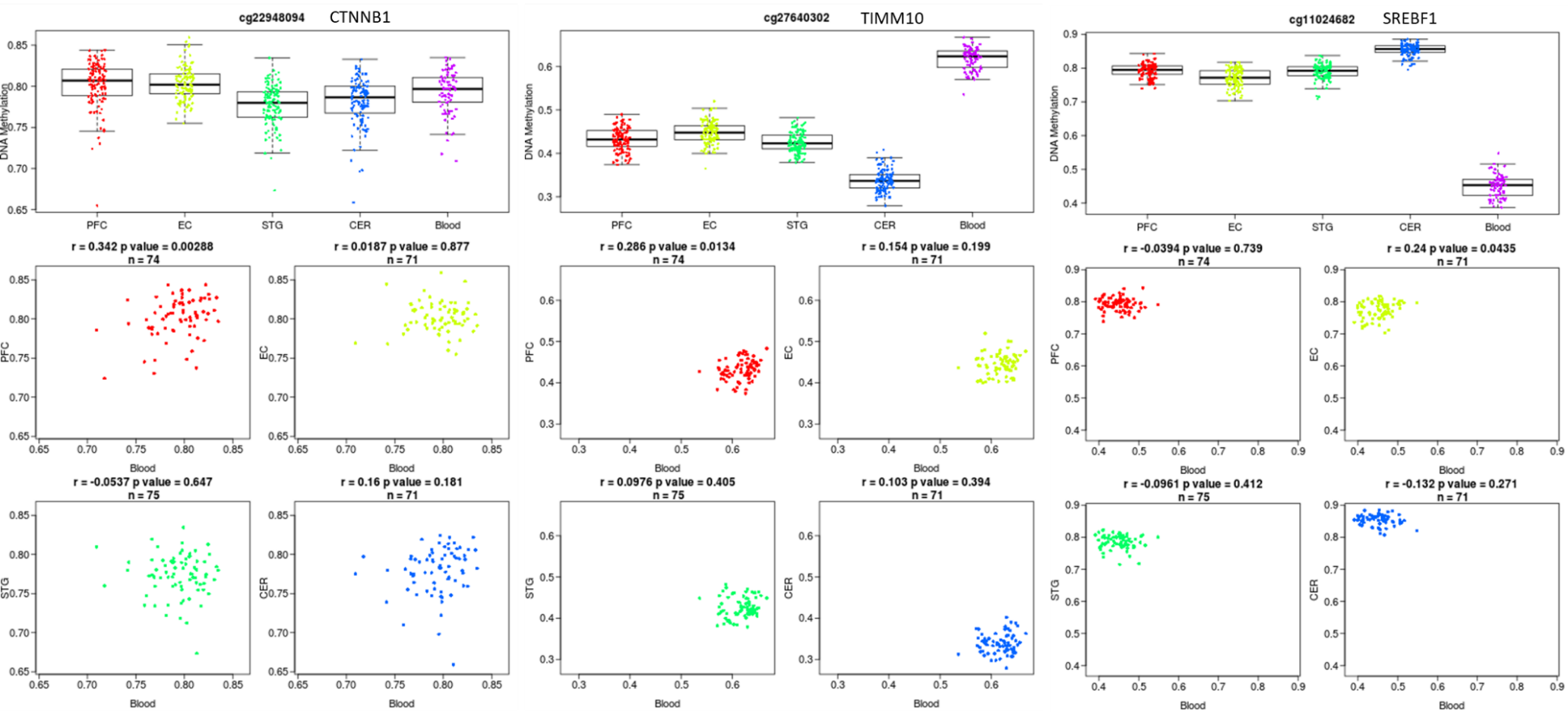
**

71-75 matched samples, DNA methylation in blood & four brain regions (prefrontal cortex, entorhinal cortex, superior temporal gyrus and cerebellum), all probes present on the Illumina 450K Beadchip array

Entorhinal cortex (EC), prefrontal cortex (PFC), superior temporal gyrus (STG), and cerebellum (CER) tissue obtained from 117 individuals archived in the MRC London Neurodegenerative Disease Brain Bank

**Supplementary Figure 5:** Look-up of the main IR-associated DNA methylation markers in six brain DNA methylomic studies of AD (Forest plots obtained from: <https://epigenetics.essex.ac.uk/shiny/MetaAna/>**,** PMID: 34112773)


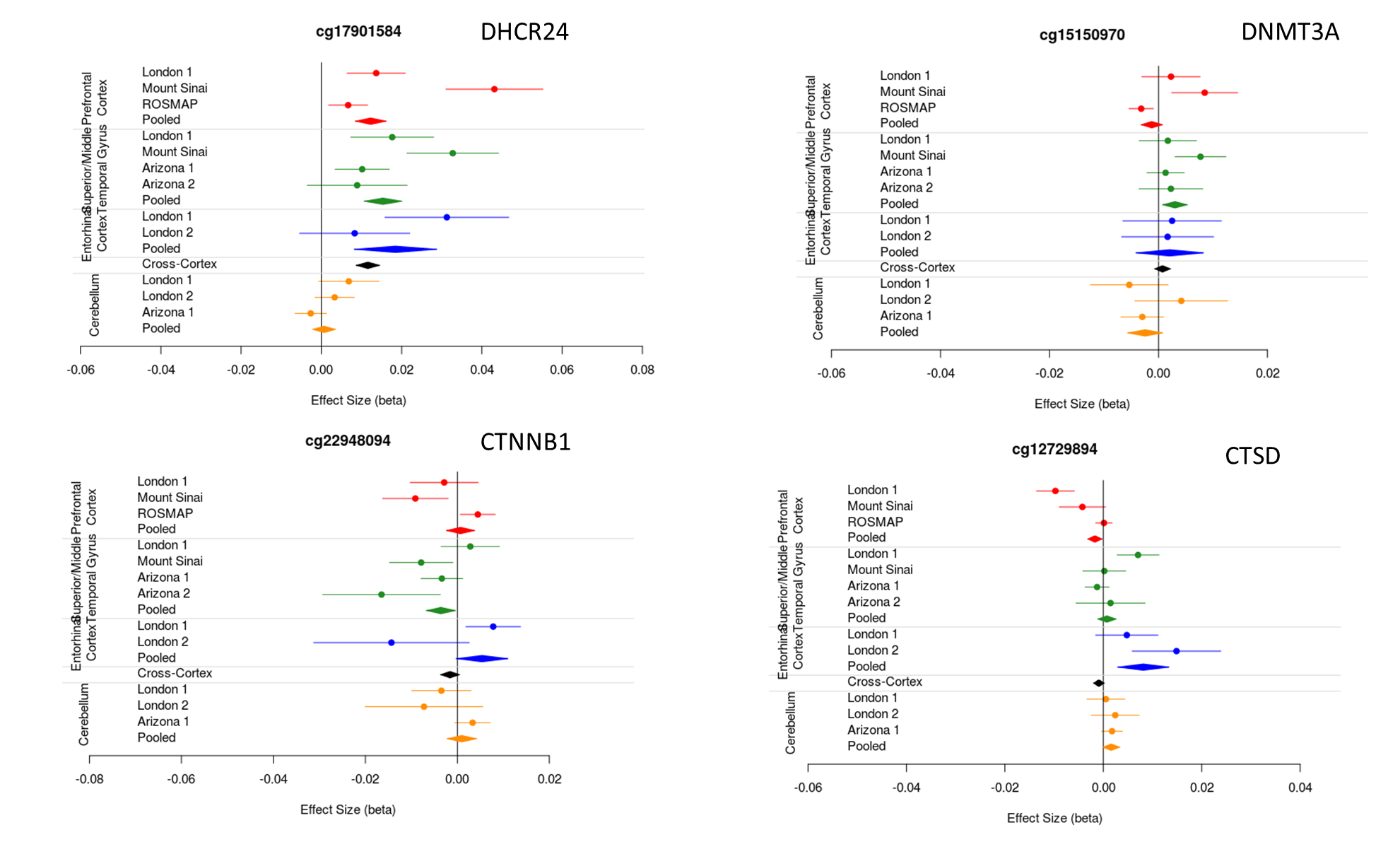


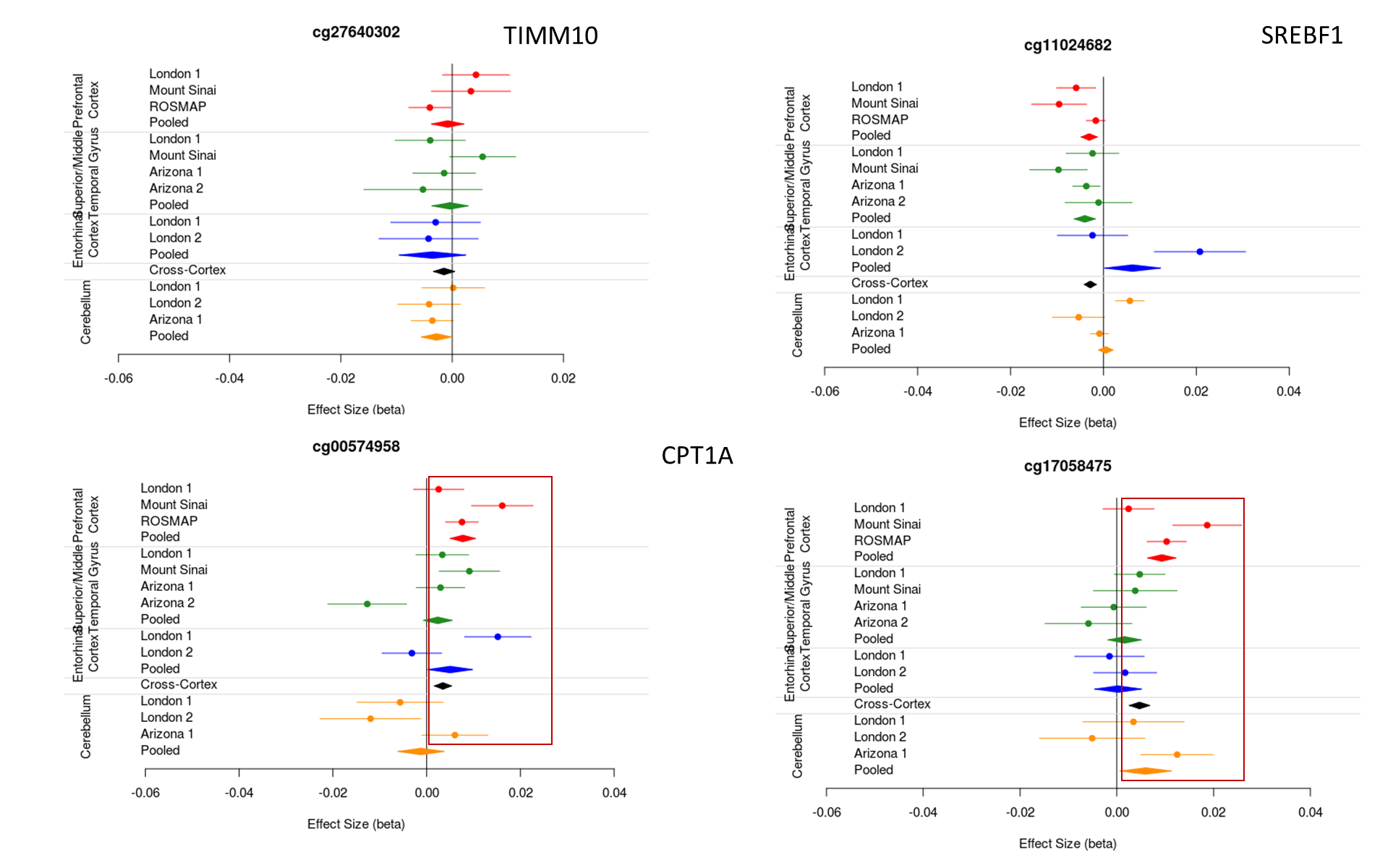


Two probes (cg06500161-*ABCG1* and cg24590708-*MYO5C*) were not present in the summary statistics.

# **Supplementary Tables**

**Supplementary Table 1**: Main blood DNA methylation markers associated (P<10^-5^) with insulin resistance, quantified using HOMA-IR, in the Framingham Heart Study (FHS)

|  |  |  |  |  | **Offspring UMN (N=1,833)** | | | **Offspring JHU (N=367)** | | | **Gen3 (N=967)** | | |
| --- | --- | --- | --- | --- | --- | --- | --- | --- | --- | --- | --- | --- | --- |
| **Locus** | **CpG marker** | **Chr** | **b37 Pos** | **Closest Gene**  **(bp distance)** | **B** | **SE** | **P** | **B** | **SE** | **P** | **B** | **SE** | **P** |
| 1p32 | cg17901584 | 1 | 55,353,706 | *DHCR24 (785)* | -0.005 | 0.0009 | ***5.4E-08*** | -0.005 | 0.0022 | 0.03 | -0.004 | 0.0015 | 0.02 |
| 2p23 | cg15150970 | 2 | 25,473,529 | *DNMT3A (0)* | 0.002 | 0.0005 | 2.3E-05 | 0.005 | 0.0029 | 0.09 | - | - | - |
| 3p22 | cg22948094 | 3 | 41,172,376 | *CTNNB1 (64025)* | -0.004 | 0.0010 | 1.5E-04 | - | - | - | -0.004 | 0.0017 | 0.02 |
| 11p15 | cg12729894 | 11 | 1,774,378 | *MOB2/CTSD (0)* | -0.004 | 0.0008 | 1.6E-05 | -0.0006 | 0.0021 | 0.76 | -0.002 | 0.0010 | 0.03 |
| 11q12 | cg27640302 | 11 | 57,296,133 | *TIMM10 (0)* | 0.002 | 0.0004 | 1.2E-04 | 0.003 | 0.0026 | 0.27 | 0.002 | 0.0010 | 0.02 |
| 11q13 | cg00574958 | 11 | 68,607,622 | *CPT1A (0)* | -0.002 | 0.0004 | ***1.7E-09*** | *-* | - | *-* | -0.002 | 0.0006 | 1.7E-04 |
| 11q13 | cg17058475 | 11 | 68,607,737 | *CPT1A (0)* | -0.003 | 0.0005 | 2.8E-06 | -0.001 | 0.0014 | 0.34 | -0.003 | 0.0011 | 0.003 |
| 15q21 | cg24590708 | 15 | 52,554,357 | *MYO5C (0)* | 0.004 | 0.0014 | 0.01 | 0.011 | 0.0034 | 0.002 | 0.005 | 0.0019 | 0.008 |
| 17p11 | cg11024682 | 17 | 17,730,094 | *SREBF1 (0)* | 0.002 | 0.0004 | 3.8E-06 | 0.001 | 0.0023 | 0.62 | 0.0008 | 0.0006 | 0.19 |
| 21q22 | cg06500161 | 21 | 43,656,587 | *ABCG1 (0)* | 0.004 | 0.0005 | ***5.1E-13*** | 0.010 | 0.0029 | 9.0E-04 | 0.003 | 0.0009 | 0.003 |

Offspring University of Minnesota Biomedical Genomics Center (UMN), Offspring Johns Hopkins Center for Inherited Disease Research (JHU), and Gen3 denote the three batches used to measure blood DNA methylation in FHS

P-values in bold and italic passed the multiple-testing significance threshold.

Linear mixed-effects models, adjusted for age, sex, and body mass index, were used to evaluate association of blood DNA methylation (outcome) with IR.

**Supplementary Table 2**: Results of the sensitivity analyses (additional adjustment for current smoking) for the main blood DNA methylation markers associated (P<10^-5^) with insulin resistance, quantified using HOMA-IR, in the Framingham Heart Study (FHS)

|  |  |  |  | **Model adjusted for BMI** | | | **Model adjusted for BMI and current smoking** | | |
| --- | --- | --- | --- | --- | --- | --- | --- | --- | --- |
| **CpG marker** | **Chr** | **b37 Pos** | **Closest Gene**  **(bp distance)** | **B** | **SE** | **P** | **B** | **SE** | **P** |
| cg17901584 | 1 | 55,353,706 | *DHCR24 (785)* | -0.0047 | 0.0007 | ***3.0E-10*** | -0.0048 | 0.0007 | ***1.7E-10*** |
| cg15150970 | 2 | 25,473,529 | *DNMT3A (0)* | 0.0023 | 0.0005 | 7.8E-06 | 0.0023 | 0.0005 | 7.8E-06 |
| cg22948094 | 3 | 41,172,376 | *CTNNB1 (64025)* | -0.0038 | 0.0009 | 7.5E-06 | -0.0038 | 0.0009 | 7.3E-06 |
| cg12729894 | 11 | 1,774,378 | *MOB2/CTSD (0)* | -0.0028 | 0.0006 | 3.6E-06 | -0.0028 | 0.0006 | 2.8E-06 |
| cg27640302 | 11 | 57,296,133 | *TIMM10 (0)* | 0.0018 | 0.0004 | 4.0E-06 | 0.0018 | 0.0004 | 3.9E-06 |
| cg00574958 | 11 | 68,607,622 | *CPT1A (0)* | -0.0024 | 0.0003 | ***1.2E-12*** | -0.0024 | 0.0003 | ***1.2E-12*** |
| cg17058475 | 11 | 68,607,737 | *CPT1A (0)* | -0.0025 | 0.0005 | ***3.7E-08*** | -0.0025 | 0.0005 | ***3.7E-08*** |
| cg24590708 | 15 | 52,554,357 | *MYO5C (0)* | 0.0048 | 0.0011 | 9.1E-06 | 0.0048 | 0.0011 | 8.1E-06 |
| cg11024682 | 17 | 17,730,094 | *SREBF1 (0)* | 0.0016 | 0.0003 | 4.7E-06 | 0.0016 | 0.0003 | 4.5E-06 |
| cg06500161 | 21 | 43,656,587 | *ABCG1 (0)* | 0.0035 | 0.0004 | ***3.4E-16*** | 0.0036 | 0.0004 | ***2.8E-16*** |

P-values in bold and italic passed the multiple-testing significance threshold.

Analyses were performed using linear mixed-effects models, adjusted for age, sex, body mass index, and +/- current smoking, to evaluate association of blood DNA methylation (outcome) with IR.

**Supplementary Table 3**: Results of the sensitivity analyses (additional adjustment for blood cell counts) for the main blood DNA methylation markers associated (P<10^-5^) with insulin resistance, quantified using HOMA-IR, in the Framingham Heart Study (FHS)

|  |  |  |  | **Model not adjusted for blood cell counts** | | | **Model adjusted for blood cell counts** | | |
| --- | --- | --- | --- | --- | --- | --- | --- | --- | --- |
| **CpG marker** | **Chr** | **b37 Pos** | **Closest Gene**  **(bp distance)** | **B** | **SE** | **P** | **B** | **SE** | **P_bacon_** |
| cg17901584 | 1 | 55,353,706 | *DHCR24 (785)* | -0.0047 | 0.0007 | ***3.0E-10*** | -0.0041 | 0.0007 | ***1.4E-08*** |
| cg15150970 | 2 | 25,473,529 | *DNMT3A (0)* | 0.0023 | 0.0005 | 7.8E-06 | 0.0020 | 0.0005 | 1.5E-04 |
| cg22948094 | 3 | 41,172,376 | *CTNNB1 (64025)* | -0.0038 | 0.0009 | 7.5E-06 | -0.0039 | 0.0008 | 2.2E-06 |
| cg12729894 | 11 | 1,774,378 | *MOB2/CTSD (0)* | -0.0028 | 0.0006 | 3.6E-06 | -0.0029 | 0.0006 | 2.7E-06 |
| cg27640302 | 11 | 57,296,133 | *TIMM10 (0)* | 0.0018 | 0.0004 | 4.0E-06 | 0.0021 | 0.0004 | 6.1E-07 |
| cg00574958 | 11 | 68,607,622 | *CPT1A (0)* | -0.0024 | 0.0003 | ***1.2E-12*** | -0.0022 | 0.0003 | ***1.3E-12*** |
| cg17058475 | 11 | 68,607,737 | *CPT1A (0)* | -0.0025 | 0.0005 | ***3.7E-08*** | -0.0023 | 0.0004 | ***2.5E-08*** |
| cg24590708 | 15 | 52,554,357 | *MYO5C (0)* | 0.0048 | 0.0011 | 9.1E-06 | 0.0046 | 0.0010 | 1.3E-05 |
| cg11024682 | 17 | 17,730,094 | *SREBF1 (0)* | 0.0016 | 0.0003 | 4.7E-06 | 0.0015 | 0.0003 | 2.1E-06 |
| cg06500161 | 21 | 43,656,587 | *ABCG1 (0)* | 0.0035 | 0.0004 | ***3.4E-16*** | 0.0036 | 0.0004 | ***8.9E-18*** |

Linear mixed-effects models, adjusted for age, sex, body mass index, and +/- blood cell counts were used to evaluate association of blood DNA methylation (outcome) with IR.

bacon R package (18) was used to remove inflation and bias in the EWAS of IR adjusted for blood cell counts

We used measured blood cell counts when available (Gen 3) and imputed them when not (Offspring), based on raw DNA methylation data.

P-values in bold and italic passed the multiple-testing significance threshold.

**Supplementary Table 4**: Sex-stratified association analysis of DNA methylation with IR for the top DNA methylation markers in FHS

|  |  | **MALE (N=1,410)** | | | **FEMALE (N=1,757)** | | |
| --- | --- | --- | --- | --- | --- | --- | --- |
| CpG marker | Closest Gene | B | SE | P | B | SE | P |
| cg17901584 | *DHCR24* | -0.0034 | 0.0011 | 2.4E-03 | -0.0058 | 0.0010 | ***9.2E-09*** |
| cg15150970 | *DNMT3A* | 0.0027 | 0.0008 | 7.5E-04 | 0.0020 | 0.0007 | 3.5E-03 |
| cg22948094 | *CTNNB1* | -0.0049 | 0.0013 | 1.3E-04 | -0.0030 | 0.0011 | 9.1E-03 |
| cg12729894 | *MOB2/CTSD* | -0.0032 | 0.0009 | 3.1E-04 | -0.0027 | 0.0008 | 1.6E-03 |
| cg27640302 | *TIMM10* | 0.0019 | 0.0006 | 2.7E-03 | 0.0018 | 0.0005 | 7.5E-04 |
| cg00574958 | *CPT1A* | -0.0021 | 0.0005 | 5.0E-05 | -0.0025 | 0.0004 | ***1.1E-08*** |
| cg17058475 | *CPT1A* | -0.0020 | 0.0007 | 4.7E-03 | -0.0029 | 0.0006 | 2.1E-06 |
| cg24590708 | *MYO5C* | 0.0025 | 0.0015 | 9.7E-02 | 0.0068 | 0.0015 | 7.8E-06 |
| cg11024682 | *SREBF1* | 0.0008 | 0.0005 | 1.0E-01 | 0.0022 | 0.0005 | 2.1E-06 |
| cg06500161 | *ABCG1* | 0.0030 | 0.0006 | 4.2E-06 | 0.0039 | 0.0006 | ***3.2E-11*** |

**Supplementary Table 5**: Sex-stratified association analysis of DNA methylation with risk of Alzheimer’s dementia for the top DNA methylation markers in FHS

|  |  | **MALE (N=938)** | | | **FEMALE (N=1,249)** | | |
| --- | --- | --- | --- | --- | --- | --- | --- |
| CpG marker | Closest Gene | B | SE | P | B | SE | P |
| cg17901584 | *DHCR24* | -0.0034 | 0.0039 | 0.383 | -0.0035 | 0.0028 | 0.216 |
| cg15150970 | *DNMT3A* | -0.0090 | 0.0027 | ***7.0E-04*** | -0.0019 | 0.0017 | 0.253 |
| cg22948094 | *CTNNB1* | -0.0001 | 0.0052 | 0.983 | 0.0032 | 0.0032 | 0.313 |
| cg12729894 | *MOB2/CTSD* | -0.0066 | 0.0032 | 0.042 | -0.0043 | 0.0023 | 0.069 |
| cg27640302 | *TIMM10* | -0.0026 | 0.0023 | 0.273 | 0.0009 | 0.0014 | 0.515 |
| cg00574958 | *CPT1A* | 0.0013 | 0.0024 | 0.589 | 0.0016 | 0.0013 | 0.222 |
| cg17058475 | *CPT1A* | 0.0026 | 0.0024 | 0.272 | 0.0023 | 0.0015 | 0.135 |
| cg24590708 | *MYO5C* | -0.0090 | 0.0056 | 0.111 | -0.0048 | 0.0042 | 0.251 |
| cg11024682 | *SREBF1* | -0.0044 | 0.0022 | 0.047 | -0.0009 | 0.0014 | 0.505 |
| cg06500161 | *ABCG1* | -0.0039 | 0.0025 | 0.125 | -0.0020 | 0.0017 | 0.229 |

P-values in bold and italic passed the multiple-testing significance threshold.

**Supplementary Table 6**: Sex-stratified association analysis of DNA methylation with brain volumes for the top DNA methylation markers in FHS

|  |  | **MALE (N=1,013)** | | | **FEMALE (N=1,274)** | | |
| --- | --- | --- | --- | --- | --- | --- | --- |
| **Hippocampal Volume** | | | | | | | |
| CpG marker | Closest Gene | B | SE | P | B | SE | P |
| cg17901584 | *DHCR24* | -0.903 | 1.464 | 0.537 | -0.704 | 1.231 | 0.567 |
| cg15150970 | *DNMT3A* | 1.148 | 1.019 | 0.260 | 0.983 | 0.792 | 0.214 |
| cg22948094 | *CTNNB1* | 0.597 | 1.577 | 0.705 | -2.819 | 1.363 | 0.039 |
| cg12729894 | *MOB2/CTSD* | 0.006 | 1.108 | 0.996 | 1.104 | 0.991 | 0.265 |
| cg27640302 | *TIMM10* | -0.207 | 0.763 | 0.786 | 0.880 | 0.629 | 0.162 |
| cg00574958 | *CPT1A* | -1.002 | 0.671 | 0.136 | 0.258 | 0.555 | 0.641 |
| cg17058475 | *CPT1A* | -0.696 | 0.900 | 0.439 | 0.809 | 0.698 | 0.246 |
| cg24590708 | *MYO5C* | -0.497 | 1.973 | 0.801 | 1.773 | 1.811 | 0.328 |
| cg11024682 | *SREBF1* | -0.807 | 0.697 | 0.247 | 0.749 | 0.578 | 0.195 |
| cg06500161 | *ABCG1* | -0.630 | 0.832 | 0.449 | 1.692 | 0.728 | 0.020 |
| **Total Brain Volume** | | | | | | | |
| CpG marker | Closest Gene | B | SE | P | B | SE | P |
| cg17901584 | *DHCR24* | -0.042 | 0.031 | 0.170 | -0.007 | 0.026 | 0.784 |
| cg15150970 | *DNMT3A* | 0.036 | 0.021 | 0.089 | 0.014 | 0.016 | 0.401 |
| cg22948094 | *CTNNB1* | 0.023 | 0.034 | 0.499 | -0.035 | 0.028 | 0.210 |
| cg12729894 | *MOB2/CTSD* | -0.004 | 0.024 | 0.866 | 0.042 | 0.021 | 0.045 |
| cg27640302 | *TIMM10* | -0.019 | 0.016 | 0.247 | 0.031 | 0.013 | 0.018 |
| cg00574958 | *CPT1A* | 0.024 | 0.015 | 0.102 | 0.018 | 0.011 | 0.113 |
| cg17058475 | *CPT1A* | 0.029 | 0.019 | 0.126 | 0.040 | 0.014 | 0.006 |
| cg24590708 | *MYO5C* | 0.008 | 0.042 | 0.847 | 0.003 | 0.038 | 0.938 |
| cg11024682 | *SREBF1* | -0.027 | 0.015 | 0.074 | 0.019 | 0.012 | 0.110 |
| cg06500161 | *ABCG1* | -0.003 | 0.018 | 0.873 | 0.003 | 0.015 | 0.867 |
| **Lateral Ventricular Volume** | | | | | | | |
| CpG marker | Closest Gene | B | SE | P | B | SE | P |
| cg17901584 | *DHCR24* | 0.002 | 0.002 | 0.314 | 0.0002 | 0.001 | 0.860 |
| cg15150970 | *DNMT3A* | -0.002 | 0.001 | 0.155 | -0.001 | 0.001 | 0.195 |
| cg22948094 | *CTNNB1* | -0.005 | 0.002 | 0.006 | 0.0007 | 0.001 | 0.645 |
| cg12729894 | *MOB2/CTSD* | 0.0004 | 0.001 | 0.736 | -0.002 | 0.001 | 0.037 |
| cg27640302 | *TIMM10* | 0.001 | 0.001 | 0.197 | -0.0009 | 0.001 | 0.168 |
| cg00574958 | *CPT1A* | -0.00004 | 0.001 | 0.961 | -0.001 | 0.001 | 0.019 |
| cg17058475 | *CPT1A* | -0.001 | 0.001 | 0.441 | -0.001 | 0.001 | 0.052 |
| cg24590708 | *MYO5C* | -0.002 | 0.002 | 0.310 | 0.0005 | 0.002 | 0.800 |
| cg11024682 | *SREBF1* | 0.0003 | 0.001 | 0.720 | -0.001 | 0.001 | 0.047 |
| cg06500161 | *ABCG1* | -0.0002 | 0.001 | 0.847 | -0.0002 | 0.001 | 0.831 |

**Supplementary Table 7**: Sex-stratified association analysis of DNA methylation with neurological traits for the top DNA methylation markers in ROSMAP

|  | **MALE (N=259)** | | | **FEMALE (N=447)** | | |
| --- | --- | --- | --- | --- | --- | --- |
| cg22948094 / *CTNNB1* | B | SE | P | B | SE | P |
| Clinical diagnosis of cognitive status at time of death | 0.0018 | 0.0016 | 0.274 | 0.0018 | 0.0016 | 0.274 |
| Braak stage | 0.0007 | 0.0020 | 0.737 | 0.0020 | 0.0016 | 0.194 |
| Cerad score | 0.00002 | 0.0022 | 0.994 | -0.0028 | 0.0015 | 0.074 |
| cg00574958 / *CPT1A* |  |  |  |  |  |  |
| Clinical diagnosis of cognitive status at time of death | 0.0014 | 0.0007 | 0.037 | 0.0012 | 0.0006 | 0.045 |
| Braak stage | 0.0014 | 0.0008 | 0.090 | 0.0019 | 0.0008 | 0.012 |
| Cerad score | -0.0017 | 0.0009 | 0.067 | -0.0025 | 0.0007 | ***9.8E-04*** |
| cg17058475 / *CPT1A* |  |  |  |  |  |  |
| Clinical diagnosis of cognitive status at time of death | 0.0003 | 0.0008 | 0.742 | 0.0018 | 0.0007 | 0.008 |
| Braak stage | 0.0016 | 0.0010 | 0.104 | 0.0016 | 0.0008 | 0.061 |
| Cerad score | -0.0018 | 0.0011 | 0.107 | -0.0022 | 0.0008 | 0.008 |

**Supplementary Table 8**: Association results of blood RNA expression with blood DNA methylation and HOMA-IR in FHS for the main genes/DNA methylation markers detected in the EWAS of blood DNA methylation with HOMA-IR

|  | **Blood DNA methylation (N=2,782)** | | | | | **HOMA-IR (N=5,088)** | | |
| --- | --- | --- | --- | --- | --- | --- | --- | --- |
| **transcript ID** | **CpG ID** | **Gene** | **B** | **SE** | **P** | **B** | **SE** | **P** |
| 3379644 | cg00574958 | *CPT1A* | -3.98 | 0.48 | 1.7E-16 | 0.024 | 0.006 | 1.9E-04 |
| 3379644 | cg17058475 | *CPT1A* | -2.05 | 0.30 | 1.2E-11 | 0.024 | 0.006 | 1.9E-04 |
| 3624513 | cg24590708 | *MYO5C* | -0.08 | 0.06 | 0.22 | 0.001 | 0.003 | 0.69 |
| 3922444 | cg06500161 | *ABCG1* | -2.39 | 0.16 | 1.2E-52 | -0.036 | 0.004 | 3.0E-22 |
| 2413907 | cg17901584 | *DHCR24* | -1.16 | 0.15 | 9.9E-15 | 0.011 | 0.005 | 0.02 |
| 2618940 | cg22948094 | *CTNNB1* | -0.11 | 0.12 | 0.33 | 0.010 | 0.004 | 0.01 |
| 2544662 | cg15150970 | *DNMT3A* | -0.36 | 0.15 | 0.01 | 0.002 | 0.003 | 0.47 |
| 3747966 | cg11024682 | *SREBF1* | -0.23 | 0.12 | 0.06 | -0.008 | 0.002 | 9.4E-04 |
| 3373946 | cg27640302 | *TIMM10* | 4.83 | 0.40 | 5.2E-33 | 0.002 | 0.009 | 0.87 |
| 3358950 | cg12729894 | *CTSD* | -0.22 | 0.17 | 0.19 | 0.002 | 0.005 | 0.65 |

Linear mixed-effects models, adjusted for age, sex, blood cell counts, and +/- body mass index were used to evaluate association of blood RNA expression (outcome) with IR or blood DNA methylation.

**Supplementary Table 9**: Association results of brain RNA expression with brain DNA methylation in ROSMAP for the nearest genes of the CpG sites identified in the EWAS of blood DNA methylation with HOMA-IR in FHS

| **ProbeID** | **TargetID** | **Gene Symbol** | **CpG ID** | **B** | **SE** | **P** |
| --- | --- | --- | --- | --- | --- | --- |
| 460593 | ILMN_1658176 | *ABCG1* | cg06500161 | -0.005 | 0.008 | 0.51 |
| 6450059 | ILMN_1695968 | *ABCG1* | cg06500161 | -0.011 | 0.021 | 0.58 |
| 4290020 | ILMN_1743638 | *ABCG1* | cg06500161 | 0.019 | 0.021 | 0.36 |
| 6060377 | ILMN_1794782 | *ABCG1* | cg06500161 | 0.004 | 0.010 | 0.67 |
| 3440292 | ILMN_2262362 | *ABCG1* | cg06500161 | -0.020 | 0.021 | 0.33 |
| 5860377 | ILMN_2329927 | *ABCG1* | cg06500161 | 0.007 | 0.009 | 0.42 |
| 2680458 | ILMN_1687589 | *CPT1A* | cg00574958 | 0.000 | 0.013 | 0.98 |
| 5290358 | ILMN_1696316 | *CPT1A* | cg00574958 | -0.010 | 0.008 | 0.25 |
| 4150091 | ILMN_1710052 | *CPT1A* | cg00574958 | -0.012 | 0.013 | 0.35 |
| 2680458 | ILMN_1687589 | *CPT1A* | cg17058475 | 0.017 | 0.016 | 0.28 |
| 5290358 | ILMN_1696316 | *CPT1A* | cg17058475 | 0.004 | 0.010 | 0.70 |
| 4150091 | ILMN_1710052 | *CPT1A* | cg17058475 | -0.041 | 0.016 | 0.009 |
| 7210095 | ILMN_1651617 | *CTNNB1* | cg22948094 | 0.016 | 0.034 | 0.64 |
| 5130681 | ILMN_1726641 | *CTNNB1* | cg22948094 | -0.094 | 0.035 | 0.007 |
| 4260379 | ILMN_1746396 | *CTNNB1* | cg22948094 | -0.008 | 0.008 | 0.33 |
| 3800671 | ILMN_1757350 | *CTNNB1* | cg22948094 | -0.011 | 0.008 | 0.18 |
| 5810184 | ILMN_1785786 | *CTNNB1* | cg22948094 | -0.031 | 0.034 | 0.36 |
| 4230376 | ILMN_1786903 | *CTNNB1* | cg22948094 | 0.009 | 0.038 | 0.81 |
| 3360253 | ILMN_1808436 | *CTNNB1* | cg22948094 | -0.033 | 0.041 | 0.42 |
| 1110092 | ILMN_1674038 | *CTSD* | cg12729894 | -0.001 | 0.004 | 0.85 |
| 4480341 | ILMN_1725510 | *DHCR24* | cg17901584 | -0.001 | 0.007 | 0.93 |
| 6450373 | ILMN_1654945 | *DNMT3A* | cg15150970 | -0.017 | 0.020 | 0.40 |
| 1300592 | ILMN_1676128 | *DNMT3A* | cg15150970 | 0.007 | 0.019 | 0.72 |
| 6130131 | ILMN_1698137 | *DNMT3A* | cg15150970 | -0.005 | 0.025 | 0.84 |
| 1980246 | ILMN_1808789 | *MYO5C* | cg24590708 | -0.007 | 0.014 | 0.62 |
| 3390343 | ILMN_1663035 | *SREBF1* | cg11024682 | 0.004 | 0.004 | 0.27 |
| 4230521 | ILMN_1695378 | *SREBF1* | cg11024682 | -0.001 | 0.020 | 0.94 |
| 6840044 | ILMN_2328986 | *SREBF1* | cg11024682 | 0.009 | 0.005 | 0.10 |
| 3310376 | ILMN_1765332 | *TIMM10* | cg27640302 | -0.016 | 0.002 | 9.6E-18 |

Linear mixed-effects models were used to evaluate association of brain DNA methylation (outcome) with brain RNA expression (N=439), adjusting for age at death, sex, substudy, race, batch, and neuronal proportions.

**Supplementary Table** **10:** Pathway analysis for blood DNA methylation markers associated with HOMA-IR at P<10^-7^

| **ONTOLOGY** | **TERM** | **N** | **DE** | **P.DE** |
| --- | --- | --- | --- | --- |
| MF | delta24(24-1) sterol reductase activity | 1 | 1 | 4.3E-04 |
| MF | carnitine O-palmitoyltransferase activity | 4 | 1 | 7.1E-04 |
| BP | amino-acid betaine metabolic process | 17 | 1 | 1.6E-03 |
| BP | steroid biosynthetic process | 172 | 2 | 2.8E-04 |
| BP | cholesterol biosynthetic process | 57 | 2 | 2.9E-05 |
| BP | carnitine shuttle | 4 | 1 | 7.1E-04 |
| BP | cholesterol metabolic process | 138 | 2 | 1.7E-04 |
| BP | carnitine metabolic process | 13 | 1 | 1.5E-03 |
| BP | negative regulation of macrophage derived foam cell differentiation | 13 | 1 | 1.9E-03 |
| BP | regulation of cholesterol esterification | 14 | 1 | 1.3E-03 |
| BP | regulation of lipid storage | 55 | 2 | 4.9E-05 |
| BP | negative regulation of cholesterol storage | 11 | 1 | 2.1E-03 |
| BP | sterol metabolic process | 153 | 2 | 2.3E-04 |
| BP | sterol biosynthetic process | 64 | 2 | 3.5E-05 |
| MF | carnitine O-acyltransferase activity | 6 | 1 | 7.1E-04 |
| MF | O-palmitoyltransferase activity | 5 | 1 | 8.7E-04 |
| MF | toxin transmembrane transporter activity | 8 | 1 | 2.4E-03 |
| BP | lipid storage | 85 | 2 | 8.6E-05 |
| BP | positive regulation of fatty acid beta-oxidation | 11 | 1 | 3.2E-03 |
| BP | intracellular lipid transport | 47 | 2 | 2.4E-05 |
| BP | cholesterol biosynthetic process via desmosterol | 4 | 1 | 9.6E-04 |
| BP | cholesterol biosynthetic process via lathosterol | 4 | 1 | 9.6E-04 |
| BP | phospholipid efflux | 13 | 1 | 2.0E-03 |
| MF | ATPase-coupled lipid transmembrane transporter activity | 8 | 1 | 2.1E-03 |
| MF | ABC-type sterol transporter activity | 2 | 1 | 6.2E-04 |
| BP | low-density lipoprotein particle remodeling | 16 | 1 | 1.8E-03 |
| BP | high-density lipoprotein particle remodeling | 16 | 1 | 2.5E-03 |
| BP | steroid esterification | 18 | 1 | 1.4E-03 |
| BP | sterol esterification | 18 | 1 | 1.4E-03 |
| BP | cholesterol esterification | 18 | 1 | 1.4E-03 |
| BP | glycoprotein transport | 3 | 1 | 1.2E-03 |
| BP | amyloid precursor protein metabolic process | 92 | 2 | 8.6E-05 |
| BP | amyloid precursor protein catabolic process | 66 | 2 | 4.2E-05 |
| BP | positive regulation of cholesterol biosynthetic process | 10 | 1 | 3.0E-03 |
| BP | alcohol biosynthetic process | 141 | 2 | 1.8E-04 |
| MF | delta24-sterol reductase activity | 1 | 1 | 4.3E-04 |
| BP | phospholipid homeostasis | 15 | 1 | 2.2E-03 |
| BP | positive regulation of small molecule metabolic process | 143 | 2 | 2.9E-04 |
| BP | cellular response to high density lipoprotein particle stimulus | 3 | 1 | 6.4E-04 |
| BP | positive regulation of cholesterol metabolic process | 12 | 1 | 3.5E-03 |
| MF | phosphatidylcholine floppase activity | 8 | 1 | 2.8E-03 |
| BP | positive regulation of sterol biosynthetic process | 10 | 1 | 3.0E-03 |
| BP | secondary alcohol metabolic process | 148 | 2 | 1.7E-04 |
| BP | secondary alcohol biosynthetic process | 57 | 2 | 2.9E-05 |
| BP | response to tetrachloromethane | 4 | 1 | 1.4E-03 |
| MF | palmitoleoyltransferase activity | 2 | 1 | 8.5E-04 |

Ontology: "BP" - biological process, "CC" - cellular component, "MF" - molecular function.

Term: GO term if testing GO pathways

N number of genes in the GO

DE number of genes that are differentially methylated

P.DE p-value for over-representation of the GO term

**Supplementary Table** **11**: Pathway analysis for blood DNA methylation markers associated with HOMA-IR at P<10^-5^

| **ONTOLOGY** | **TERM** | **N** | **DE** | **P.DE** |
| --- | --- | --- | --- | --- |
| MF | delta24(24-1) sterol reductase activity | 1 | 1 | 9.0E-04 |
| MF | DNA (cytosine-5-)-methyltransferase activity | 3 | 1 | 3.2E-03 |
| MF | carnitine O-palmitoyltransferase activity | 4 | 1 | 2.6E-03 |
| CC | TIM23 mitochondrial import inner membrane translocase complex | 11 | 1 | 2.0E-03 |
| BP | steroid biosynthetic process | 172 | 3 | 5.8E-05 |
| BP | cholesterol biosynthetic process | 57 | 3 | 1.8E-06 |
| BP | mitochondrial transport | 177 | 3 | 4.8E-05 |
| BP | carnitine shuttle | 4 | 1 | 2.6E-03 |
| BP | cholesterol metabolic process | 138 | 3 | 2.9E-05 |
| MF | DNA-methyltransferase activity | 6 | 1 | 5.7E-03 |
| BP | regulation of lipid storage | 55 | 3 | 3.5E-06 |
| BP | positive regulation of steroid biosynthetic process | 23 | 2 | 1.1E-04 |
| BP | sterol metabolic process | 153 | 3 | 4.4E-05 |
| BP | sterol biosynthetic process | 64 | 3 | 2.6E-06 |
| MF | carnitine O-acyltransferase activity | 6 | 1 | 2.9E-03 |
| MF | O-palmitoyltransferase activity | 5 | 1 | 2.8E-03 |
| MF | toxin transmembrane transporter activity | 8 | 1 | 5.5E-03 |
| BP | lipid storage | 85 | 3 | 9.7E-06 |
| BP | intracellular lipid transport | 47 | 2 | 2.6E-04 |
| BP | DNA methylation on cytosine | 6 | 1 | 5.7E-03 |
| MF | sterol response element binding | 3 | 1 | 4.6E-03 |
| MF | membrane insertase activity | 14 | 1 | 2.7E-03 |
| BP | cholesterol biosynthetic process via desmosterol | 4 | 1 | 2.3E-03 |
| BP | cholesterol biosynthetic process via lathosterol | 4 | 1 | 2.3E-03 |
| MF | ATPase-coupled lipid transmembrane transporter activity | 8 | 1 | 5.4E-03 |
| MF | ABC-type sterol transporter activity | 2 | 1 | 1.6E-03 |
| BP | glycoprotein transport | 3 | 1 | 3.1E-03 |
| CC | mitochondrial intermembrane space protein transporter complex | 6 | 1 | 1.5E-03 |
| CC | TIM22 mitochondrial import inner membrane insertion complex | 7 | 1 | 1.2E-03 |
| BP | amyloid precursor protein catabolic process | 66 | 2 | 4.5E-04 |
| BP | DNA methylation involved in embryo development | 9 | 1 | 4.5E-03 |
| BP | protein insertion into mitochondrial inner membrane | 13 | 1 | 2.8E-03 |
| BP | response to ethanol | 118 | 3 | 5.4E-05 |
| BP | regulation of cholesterol biosynthetic process | 22 | 2 | 7.9E-05 |
| BP | positive regulation of cholesterol biosynthetic process | 10 | 2 | 2.5E-05 |
| BP | positive regulation of lipid metabolic process | 153 | 3 | 1.2E-04 |
| BP | positive regulation of steroid metabolic process | 33 | 2 | 1.5E-04 |
| BP | alcohol biosynthetic process | 141 | 3 | 3.1E-05 |
| MF | delta24-sterol reductase activity | 1 | 1 | 9.0E-04 |
| BP | positive regulation of small molecule metabolic process | 143 | 3 | 6.0E-05 |
| BP | response to fatty acid | 63 | 2 | 4.9E-04 |
| BP | cellular response to fatty acid | 35 | 2 | 1.1E-04 |
| BP | cellular response to high density lipoprotein particle stimulus | 3 | 1 | 1.7E-03 |
| BP | C-5 methylation of cytosine | 5 | 1 | 3.7E-03 |
| BP | regulation of cholesterol metabolic process | 36 | 2 | 1.7E-04 |
| BP | positive regulation of cholesterol metabolic process | 12 | 2 | 3.5E-05 |
| BP | regulation of sterol biosynthetic process | 22 | 2 | 7.9E-05 |
| BP | positive regulation of sterol biosynthetic process | 10 | 2 | 2.5E-05 |
| MF | protein-cysteine methyltransferase activity | 2 | 1 | 2.2E-03 |
| BP | changes to DNA methylation involved in embryo development | 9 | 1 | 4.5E-03 |
| BP | organic hydroxy compound biosynthetic process | 240 | 3 | 1.7E-04 |
| BP | secondary alcohol metabolic process | 148 | 3 | 3.1E-05 |
| BP | secondary alcohol biosynthetic process | 57 | 3 | 1.8E-06 |
| BP | regulation of alcohol biosynthetic process | 51 | 2 | 4.7E-04 |
| BP | positive regulation of alcohol biosynthetic process | 27 | 2 | 1.3E-04 |
| BP | response to bisphenol A | 4 | 1 | 3.0E-03 |
| BP | cellular response to bisphenol A | 4 | 1 | 3.0E-03 |
| BP | response to tetrachloromethane | 4 | 1 | 4.3E-03 |
| MF | palmitoleoyltransferase activity | 2 | 1 | 2.1E-03 |

Ontology: "BP" - biological process, "CC" - cellular component, "MF" - molecular function.

Term: GO term if testing GO pathways

N number of genes in the GO

DE number of genes that are differentially methylated

P.DE p-value for over-representation of the GO term

**Supplementary Table 12**: Cis-methylation quantitative trait loci (mQTLs) reported at FDR<0.05 using BIOS QTL Browser (whole blood, <https://molgenis26.gcc.rug.nl/downloads/biosqtlbrowser/>) for the top blood DNA methylation markers associated with HOMA-IR

| **Pvalue** | **SNP** | **SNP Chr** | **SNP Pos** | **Probe** | **Gene** | **Probe Chr** | **Probe Pos** | **Alleles** | **Allele Assessed** | **Overall Zscore** |
| --- | --- | --- | --- | --- | --- | --- | --- | --- | --- | --- |
| 6.22E-33 | rs687565 | 1 | 55364663 | cg17901584 | *DHCR24* | 1 | 55353730 | C/A | C | 11.95 |
| 4.79E-54 | rs11681447 | 2 | 25472784 | cg15150970 | *DNMT3A* | 2 | 25473505 | T/C | C | -15.48 |
| 8.96E-19 | rs11694842 | 2 | 25482970 | cg15150970 | *DNMT3A* | 2 | 25473505 | A/G | G | 8.85 |
| 7.12E-11 | rs3754861 | 2 | 25393722 | cg15150970 | *DNMT3A* | 2 | 25473505 | A/C | C | 6.52 |
| 1.16E-10 | rs55896493 | 2 | 25498565 | cg15150970 | *DNMT3A* | 2 | 25473505 | C/G | G | 6.44 |
| 3.36E-07 | rs116734066 | 2 | 25262928 | cg15150970 | *DNMT3A* | 2 | 25473505 | C/T | T | -5.10 |
| 7.13E-41 | rs12214 | 11 | 1774666 | cg12729894 | *CTSD* | 11 | 1774354 | T/C | C | -13.39 |
| 8.35E-16 | rs10839641 | 11 | 1803095 | cg12729894 | *CTSD* | 11 | 1774354 | C/T | T | 8.05 |
| 8.92E-08 | rs2063551 | 11 | 1855854 | cg12729894 | *CTSD* | 11 | 1774354 | G/C | G | 5.35 |
| 1.31E-06 | rs187633053 | 11 | 1796580 | cg12729894 | *CTSD* | 11 | 1774354 | A/G | G | 4.84 |
| 2.43E-05 | rs35640004 | 11 | 1779768 | cg12729894 | *CTSD* | 11 | 1774354 | C/A | A | 4.22 |
| 4.38E-51 | rs2649665 | 11 | 57289328 | cg27640302 | *TIMM10* | 11 | 57296109 | C/A | A | 15.03 |
| 3.27E-310 | rs10896623 | 11 | 57295864 | cg27640302 | *TIMM10* | 11 | 57296109 | G/A | G | -41.10 |
| 4.83E-19 | rs8070432 | 17 | 17480474 | cg11024682 | *SREBF1* | 17 | 17730070 | T/C | C | 8.92 |

**Supplementary Table 13**: Cis-methylation quantitative trait loci (mQTLs) reported significant after Bonferroni correction using Brain xQTLServe (brain, <https://mostafavilab.stat.ubc.ca/xqtl/>) for the main blood DNA methylation markers associated with HOMA-IR

| **SNP** | **Chromosome** | **SNPpos** | **chromHMMlabel** | **Probe** | **Probe Chromosome** | **Probe Position Start** | **Spearman Rho** | **Pvalue** |
| --- | --- | --- | --- | --- | --- | --- | --- | --- |
| rs4938869 | 11 | 57291801 | TxWk | cg27640302 | 11 | 57296133 | 0.392 | 1.3E-18 |
| rs7925116 | 11 | 57291843 | TxWk | cg27640302 | 11 | 57296133 | 0.583 | <1E-30 |
| rs4939149 | 11 | 57293538 | TxWk | cg27640302 | 11 | 57296133 | 0.392 | 1.3E-18 |
| rs10896623 | 11 | 57295864 | Tx | cg27640302 | 11 | 57296133 | 0.583 | <1E-30 |
| rs10792101 | 11 | 57296659 | Tx | cg27640302 | 11 | 57296133 | 0.392 | 1.3E-18 |
| rs17453436 | 11 | 57297271 | Enh | cg27640302 | 11 | 57296133 | 0.337 | 7.1E-14 |
| chr11.57300982 | 11 | 57300982 | Quies | cg27640302 | 11 | 57296133 | 0.337 | 7.1E-14 |
| rs881395 | 21 | 43652410 | Enh | cg06500161 | 21 | 43656587 | 0.277 | 1.1E-09 |
| rs915846 | 21 | 43652782 | Enh | cg06500161 | 21 | 43656587 | 0.270 | 3.0E-09 |
| rs225440 | 21 | 43653053 | Enh | cg06500161 | 21 | 43656587 | 0.267 | 4.4E-09 |
| rs225441 | 21 | 43653200 | Enh | cg06500161 | 21 | 43656587 | 0.267 | 4.4E-09 |
| rs9984192 | 21 | 43654061 | Enh | cg06500161 | 21 | 43656587 | -0.424 | 6.9E-22 |
| rs4148114 | 21 | 43655267 | TssAFlnk | cg06500161 | 21 | 43656587 | 0.295 | 7.5E-11 |
| rs9982016 | 21 | 43656594 | TxWk | cg06500161 | 21 | 43656587 | -0.424 | 6.9E-22 |
| rs915847 | 21 | 43657248 | Enh | cg06500161 | 21 | 43656587 | 0.302 | 2.7E-11 |
| rs9983344 | 21 | 43657996 | TxWk | cg06500161 | 21 | 43656587 | -0.424 | 6.9E-22 |
| rs225443 | 21 | 43658206 | TxWk | cg06500161 | 21 | 43656587 | 0.299 | 3.7E-11 |
| rs28510925 | 21 | 43659089 | TxWk | cg06500161 | 21 | 43656587 | -0.424 | 6.9E-22 |
| rs225448 | 21 | 43659830 | Enh | cg06500161 | 21 | 43656587 | 0.286 | 2.9E-10 |
| rs9978671 | 21 | 43660279 | Quies | cg06500161 | 21 | 43656587 | -0.424 | 6.9E-22 |
| rs178743 | 21 | 43660591 | Quies | cg06500161 | 21 | 43656587 | 0.302 | 2.7E-11 |
| chr21.43660688 | 21 | 43660688 | Quies | cg06500161 | 21 | 43656587 | -0.424 | 6.9E-22 |
| rs170445 | 21 | 43661486 | Quies | cg06500161 | 21 | 43656587 | 0.305 | 1.6E-11 |

mQTLs declared significant after Bonferroni correction, and computed on 468 individuals from ROSMAP and 420103 CpG sites

**Supplementary Table 14**: Cis-methylation quantitative trait loci (mQTLs) reported using GoDMC (whole blood, <http://mqtldb.godmc.org.uk/index.php>) for the main blood DNA methylation markers associated with HOMA-IR

| **rsid** | **a1** | **a2** | **name** | **CpG** | **beta_a1** | **se** | **Sample size** | **pval** | **hetisq** |
| --- | --- | --- | --- | --- | --- | --- | --- | --- | --- |
| rs4927176 | C | T | chr1:55354335:SNP | cg17901584 | 0.18779 | 0.00855 | 27749 | 7.6E-107 | 32.2 |
| chr2:25485662:D | D | I | chr2:25485662:INDEL | cg15150970 | 0.23051 | 0.00925 | 23192 | 5.1E-137 | 70.8 |
| rs62142312 | C | T | chr2:24868816:SNP | cg15150970 | 0.27555 | 0.04465 | 7825 | <1E-145 | 59.8 |
| rs12214 | C | T | chr11:1774666:SNP | cg12729894 | -0.3098 | 0.01364 | 13540 | 3.3E-114 | 44.1 |
| rs2029463 | T | C | chr11:1661905:SNP | cg12729894 | 0.10102 | 0.01289 | 26280 | <1E-145 | 44.5 |
| rs1615585 | T | C | chr11:57473675:SNP | cg27640302 | -0.1855 | 0.01107 | 27254 | 4.4E-63 | 29.4 |
| rs2581921 | C | G | chr11:57222607:SNP | cg27640302 | 0.45155 | 0.01036 | 23518 | <1E-145 | 78.1 |
| rs11652574 | A | G | chr17:17728815:SNP | cg11024682 | 1.09549 | 0.02756 | 19085 | <1E-145 | 66.8 |
| rs220182 | C | T | chr21:43561732:SNP | cg06500161 | -0.0608 | 0.00909 | 24474 | <1E-145 | 38.2 |

Only cis and clumped results were selected

**Supplementary Table 15:** Selection of cis-methylation quantitative trait loci (mQTLs) reported in FHS (whole blood) for the main blood DNA methylation markers associated with HOMA-IR (PMID: 36380121)

| **SNP (chr:pos:alleles:rsid)** | **Effect allele frequency** | **CpG** | **CpG_Chr** | **CpG_Pos** | **Gene** | **Beta** | **P** |
| --- | --- | --- | --- | --- | --- | --- | --- |
| chr1:54887409:C:G:rs6681644 | 0.56 | cg17901584 | 1 | 54888032 | *DHCR24* | -0.006 | 1.3E-14 |
| chr1:54912567:T:TAAATAAAATA:rs373443324 | 0.37 | cg17901584 | 1 | 54888032 | *DHCR24* | -0.007 | 3.1E-11 |
| chr1:54815458:TCC:T:rs145750607 | 0.60 | cg17901584 | 1 | 54888032 | *DHCR24* | -0.004 | 5.2E-08 |
| chr11:57528391:A:G:rs10896623 | 0.35 | cg27640302 | 11 | 57528659 | *TIMM10* | -0.015 | 0.0E+00 |
| chr11:57585182:T:G:rs7112126 | 0.24 | cg27640302 | 11 | 57528659 | *TIMM10* | -0.013 | 1.4E-162 |
| chr11:57527192:TG:T:rs144424694 | 0.12 | cg27640302 | 11 | 57528659 | *TIMM10* | -0.013 | 2.7E-93 |
| chr11:1753436:T:C:rs12214 | 0.29 | cg12729894 | 11 | 1753147 | *CTSD* | -0.007 | 6.4E-55 |
| chr11:1724780:G:A:rs72850947 | 0.08 | cg12729894 | 11 | 1753147 | *CTSD* | -0.010 | 1.3E-34 |
| chr11:1735618:C:T:rs4752755 | 0.49 | cg12729894 | 11 | 1753147 | *CTSD* | 0.005 | 1.4E-28 |
| chr11:1778383:G:A:rs145507593 | 0.02 | cg12729894 | 11 | 1753147 | *CTSD* | -0.013 | 8.4E-16 |
| chr11:1784725:G:A:rs10839657 | 0.35 | cg12729894 | 11 | 1753147 | *CTSD* | 0.003 | 2.2E-14 |
| chr11:68837155:T:C:rs144276682 | 0.01 | cg17058475 | 11 | 68840268 | *CPT1A* | 0.011 | 7.3E-10 |
| chr11:68956187:A:G:rs911606944 | 0.04 | cg00574958 | 11 | 68840153 | *CPT1A* | 0.004 | 7.7E-07 |
| chr17:17826717:C:A:rs35367147 | 0.03 | cg11024682 | 17 | 17826779 | *SREBF1* | 0.023 | 6.7E-74 |
| chr17:17832638:T:C:rs4924821 | 0.59 | cg11024682 | 17 | 17826779 | *SREBF1* | -0.005 | 6.8E-33 |
| chr17:18000077:CGT:C:rs551086311 | 0.19 | cg11024682 | 17 | 17826779 | *SREBF1* | 0.006 | 1.1E-26 |
| chr2:25251226:G:A:rs184800959 | 0.03 | cg15150970 | 2 | 25250659 | *DNMT3A* | 0.019 | 1.5E-67 |
| chr2:25250678:A:T:rs747792 | 0.69 | cg15150970 | 2 | 25250659 | *DNMT3A* | 0.006 | 2.8E-57 |
| chr2:25234839:T:A:rs11695471 | 0.32 | cg15150970 | 2 | 25250659 | *DNMT3A* | 0.005 | 1.4E-40 |
| chr2:25262793:CT:C:rs34234625 | 0.46 | cg15150970 | 2 | 25250659 | *DNMT3A* | 0.005 | 4.9E-36 |
| chr21:42236484:A:T:rs9982016 | 0.04 | cg06500161 | 21 | 42236476 | *ABCG1* | 0.011 | 2.8E-15 |
| chr21:42238096:G:A:rs225443 | 0.37 | cg06500161 | 21 | 42236476 | *ABCG1* | -0.004 | 4.2E-11 |

A total of 9,607 cis-mQTLs with a minor effect allele frequency ≥1% were reported at P≤5E-6

**Supplementary Table 16:** Association results of blood RNA gene expression (RNA-seq) (19) with blood DNA methylation for the main genes/DNA methylation markers detected in the EWAS of blood DNA methylation with HOMA-IR from an expression quantitative trait methylation analysis conducted in FHS

| **CpG** | **CpG**  **Gene Symbol** | **Tx** | **Tx_Chr** | **Tx_Start** | **Tx_Gene** | **Fx** | **T** | **P** |
| --- | --- | --- | --- | --- | --- | --- | --- | --- |
| cg27640302 | *TIMM10* | ENSG00000134809.9 | 11 | 57528464 | *TIMM10* | 4.62 | 13.02 | 2.7E-37 |
| cg27640302 | *TIMM10* | ENSG00000265566.2 | 11 | 57528085 | *RN7SL605P* | 0.30 | 6.50 | 1.0E-10 |
| cg00574958 | *CPT1A* | ENSG00000110090.13 | 11 | 68754620 | *CPT1A* | -3.69 | -7.08 | 2.0E-12 |
| cg17058475 | *CPT1A* | ENSG00000110090.13 | 11 | 68754620 | *CPT1A* | -2.28 | -5.75 | 1.0E-08 |
| cg11024682 | *SREBF1* | ENSG00000072310.16 | 17 | 17810399 | *SREBF1* | -2.23 | -8.35 | 1.2E-16 |
| cg11024682 | *SREBF1* | ENSG00000171953.16 | 17 | 17977409 | *ATPAF2* | -0.71 | -5.53 | 3.7E-08 |
| cg06500161 | *ABCG1* | ENSG00000160179.18 | 21 | 42199689 | *ABCG1* | -6.28 | -18.58 | 2.6E-71 |

Normalized DNA methylation β values were residualized after accounting for batch effects, row effects, column effects, and four PCs constructed from the normalized β values. Normalized gene expression value was residualized to account for batch effects, RNA concentration, and RNA integrity number. For each CpG-transcript pair, residualized gene expression was modeled as the outcome with residualized DNA methylation β values as the primary explanatory variable. Models were adjusted for age, sex, white blood cell count, blood cell fraction, platelet count, five gene expression PCs, and ten DNA methylation PCs.

**Supplementary Table 17:** Correlation of DNA methylation in blood and brain for the main DNA methylation markers identified in the EWAS of blood DNA methylation with HOMA-IR (PMID: 30705257)

Twenty-seven subjects with medically intractable epilepsy undergoing neurosurgery were recruited for this study between March 2014 and April 2017 at the University of Iowa Hospitals and Clinics.

1. 450K

|  |  |  |  | **Brain - blood** | | **Brain - saliva** | | **Blood - saliva** | |
| --- | --- | --- | --- | --- | --- | --- | --- | --- | --- |
| **CpG** | **CHR** | **MAPINFO** | **UCSC**  **RefGene Name** | **rho** | **p** | **rho** | **p** | **rho** | **p** |
| cg17901584 | 1 | 55353706 | *DHCR24* | 0.236 | 0.514 | -0.115 | 0.759 | 0.709 | 0.028 |
| cg15150970 | 2 | 25473529 | *DNMT3A* | 0.152 | 0.682 | 0.152 | 0.682 | -0.067 | 0.865 |
| cg22948094 | 3 | 41172376 |  | 0.370 | 0.296 | -0.261 | 0.470 | -0.588 | 0.080 |
| cg12729894 | 11 | 1774378 | *HCCA2;CTSD* | 0.321 | 0.368 | 0.564 | 0.096 | -0.018 | 0.973 |
| cg27640302 | 11 | 57296133 | *TIMM10* | 0.745 | 0.018 | 0.127 | 0.733 | -0.091 | 0.811 |
| cg00574958 | 11 | 68607622 | *CPT1A* | 0.018 | 0.973 | -0.212 | 0.560 | -0.224 | 0.537 |
| cg17058475 | 11 | 68607737 | *CPT1A* | -0.006 | 1.000 | -0.273 | 0.448 | 0.297 | 0.407 |
| cg24590708 | 15 | 52554357 | *MYO5C* | -0.055 | 0.892 | 0.321 | 0.368 | 0.188 | 0.608 |
| cg11024682 | 17 | 17730094 | *SREBF1* | 0.418 | 0.232 | -0.321 | 0.368 | -0.139 | 0.707 |
| cg06500161 | 21 | 43656587 | *ABCG1* | 0.164 | 0.657 | -0.127 | 0.733 | -0.297 | 0.407 |

Genome-wide DNA methylation was assessed using the Infinium HumanMethylation450 array on 12 subjects, initially, for brain, blood, and saliva samples.

1. EPIC

|  |  |  |  | **Brain - blood** | | **Brain - saliva** | | **Brain - buccal** | | **Blood - saliva** | | **Blood - buccal** | | **Saliva – buccal** | |
| --- | --- | --- | --- | --- | --- | --- | --- | --- | --- | --- | --- | --- | --- | --- | --- |
| **CpG** | **CHR** | **MAPINFO** | **UCSC**  **RefGene Name** | **rho** | **p** | **rho** | **p** | **rho** | **p** | **rho** | **p** | **rho** | **p** | **rho** | **p** |
| cg17901584 | 1 | 55353706 | *DHCR24* | 0.099 | 0.670 | -0.262 | 0.250 | -0.029 | 0.903 | 0.314 | 0.165 | 0.247 | 0.280 | 0.448 | 0.043 |
| cg15150970 | 2 | 25473529 | *DNMT3A* | 0.526 | 0.016 | -0.183 | 0.425 | 0.126 | 0.585 | 0.308 | 0.174 | 0.525 | 0.016 | 0.825 | 2.1E-06 |
| cg22948094 | 3 | 41172376 |  | 0.352 | 0.118 | 0.410 | 0.066 | 0.353 | 0.117 | 0.560 | 0.009 | 0.119 | 0.605 | 0.414 | 0.063 |
| cg12729894 | 11 | 1774378 | *HCCA2;CTSD* | 0.551 | 0.011 | 0.119 | 0.605 | 0.388 | 0.083 | 0.599 | 0.005 | 0.577 | 0.007 | 0.382 | 0.089 |
| cg27640302 | 11 | 57296133 | *TIMM10* | 0.330 | 0.144 | 0.239 | 0.295 | 0.357 | 0.113 | 0.444 | 0.045 | 0.083 | 0.720 | 0.748 | 1.5E-04 |
| cg00574958 | 11 | 68607622 | *CPT1A* | 0.134 | 0.562 | 0.057 | 0.806 | 0.210 | 0.358 | 0.403 | 0.071 | 0.548 | 0.011 | 0.738 | 2.1E-04 |
| cg17058475 | 11 | 68607737 | *CPT1A* | 0.208 | 0.364 | -0.287 | 0.206 | 0.191 | 0.405 | -0.101 | 0.661 | 0.342 | 0.130 | 0.284 | 0.211 |
| cg24590708 | 15 | 52554357 | *MYO5C* | -0.040 | 0.863 | 0.101 | 0.661 | 0.074 | 0.750 | 0.386 | 0.085 | 0.240 | 0.293 | 0.271 | 0.233 |
| cg11024682 | 17 | 17730094 | *SREBF1* | 0.462 | 0.036 | -0.325 | 0.151 | -0.370 | 0.099 | 0.017 | 0.944 | 0.153 | 0.506 | 0.895 | 2.7E-06 |
| cg06500161 | 21 | 43656587 | *ABCG1* | 0.747 | 1.5E-04 | 0.295 | 0.194 | 0.353 | 0.117 | 0.340 | 0.131 | 0.199 | 0.386 | 0.521 | 0.017 |

The Infinium HumanMethylationEPIC was used to analyze 21 subjects with brain, blood, saliva, and buccal samples, with six subjects overlapping between the two datasets (450K versus EPIC).

**Supplementary Table 18:** Look-up of the main IR-associated blood DNA methylation markers in a meta-analysis of EWAS of AD (PMID: 35982059)

1. Blood tissue meta-analysis (1284 blood samples from the ADNI (n = 793) and AIBL (n = 491) datasets)

| **CpG** | **Closest Gene** | **Beta_bacon** | **SE bacon** | **Pvalue fixed effect bacon** | **Pvalue random effect bacon** | **Cochran Pvalue Q statistic**  **bacon** | **Direction** | **Final Pvalue bacon** |
| --- | --- | --- | --- | --- | --- | --- | --- | --- |
| cg17901584 | *DHCR24* | -2.074 | 1.382 | 0.134 | 0.134 | 0.631 | -- | 0.134 |
| cg15150970 | *DNMT3A* | -4.932 | 1.943 | 0.011 | 0.048 | 0.184 | -- | 0.011 |
| cg22948094 | *CTNNB1* | -2.804 | 4.792 | 0.558 | 0.538 | 0.263 | -- | 0.558 |
| cg27640302 | *TIMM10* | -1.186 | 2.434 | 0.626 | 0.626 | 0.494 | -+ | 0.626 |
| cg00574958 | *CPT1A* | 5.423 | 4.433 | 0.221 | 0.340 | 0.185 | +- | 0.221 |
| cg17058475 | *CPT1A* | -1.936 | 3.552 | 0.586 | 0.608 | 0.004 | +- | 0.586 |
| cg24590708 | *MYO5C* | -1.672 | 1.465 | 0.254 | 0.794 | 1.2E-04 | +- | 0.254 |
| cg11024682 | *SREBF1* | -0.196 | 2.321 | 0.933 | 0.909 | 0.162 | +- | 0.933 |
| cg06500161 | *ABCG1* | 4.844 | 2.131 | 0.023 | 0.248 | 0.072 | ++ | 0.023 |

The probe cg12729894-*CTSD* was not present in the summary statistics.

1. Cross tissue meta-analysis (1284 blood samples from the ADNI (n = 793) and AIBL (n = 491) datasets, and an additional 1030 prefrontal cortex brain samples from four independent datasets, which included samples from the ROSMAP (n = 726), Mt. Sinai (n = 141), London (n = 107), and Gasparoni (n = 56) studies)

| **CpG** | **Closest Gene** | **SumZ** | **Pvalue** | **GASPARONI**  **Pvalue bacon** | **LONDON**  **Pvalue bacon** | **MTSINAI**  **Pvalue bacon** | **ROSMAP**  **Pvalue bacon** | **ADNI Pvalue**  **bacon** | **AIBL Pvalue bacon** |
| --- | --- | --- | --- | --- | --- | --- | --- | --- | --- |
| cg17901584 | *DHCR24* | 2.276 | 0.011 | 0.595 | 0.064 | 0.003 | 0.142 | 0.443 | 0.169 |
| cg15150970 | *DNMT3A* | 1.057 | 0.145 | 0.057 | 0.244 | 0.904 | 0.810 | 0.242 | 0.009 |
| cg22948094 | *CTNNB1* | -0.835 | 0.798 | 0.850 | 0.920 | 0.389 | 0.445 | 0.976 | 0.207 |
| cg27640302 | *TIMM10* | -0.413 | 0.660 | 0.087 | 0.754 | 0.649 | 0.457 | 0.412 | 0.860 |
| cg00574958 | *CPT1A* | 0.099 | 0.461 | 0.904 | 0.737 | 0.126 | 0.090 | 0.071 | 0.996 |
| cg17058475 | *CPT1A* | 3.372 | 3.7E-04 | 0.801 | 0.267 | 0.155 | 0.006 | 0.256 | 0.007 |
| cg24590708 | *MYO5C* | 1.985 | 0.024 | 0.866 | 0.494 | 0.497 | 0.667 | 0.047 | 4.7E-04 |
| cg11024682 | *SREBF1* | 0.290 | 0.386 | 0.601 | 0.351 | 0.112 | 0.804 | 0.385 | 0.272 |
| cg06500161 | *ABCG1* | -0.220 | 0.587 | 0.887 | 0.634 | 0.939 | 0.565 | 0.004 | 0.894 |

The probe cg12729894-*CTSD* was not present in the summary statisticsstylefix
